# High-fat and high-carbohydrate diets increase bone fragility through TGF-**β**–dependent control of osteocyte function

**DOI:** 10.1172/jci.insight.175103

**Published:** 2024-07-09

**Authors:** Neha S. Dole, Andrés Betancourt-Torres, Serra Kaya, Yoshihiro Obata, Charles A. Schurman, Jihee Yoon, Cristal S. Yee, Vivek Khanal, Clarissa Aguirre Luna, Madeline Carroll, Jennifer J. Salinas, Elizabeth Miclau, Claire Acevedo, Tamara Alliston

**Affiliations:** 1 Department of Orthopaedic Surgery, University of California, San Francisco, San Francisco, California, USA.; 2 Department of Physiology and Cell Biology, University of Arkansas for Medical Sciences, Little Rock Arkansas, USA.; 3 Department of Mechanical Engineering, University of Utah, Salt Lake City, Utah, USA.; 4 UC Berkeley/UCSF Graduate Program in Bioengineering, San Francisco, California, USA.; 5 Department of Biomedical Engineering, University of Utah, Salt Lake City, Utah, USA.; 6 Department of Mechanical and Aerospace Engineering, University of California, San Diego, San Diego, California, USA.

**Keywords:** Bone biology, Obesity

## Abstract

Obesity can increase the risk of bone fragility, even when bone mass is intact. This fragility stems from poor bone quality, potentially caused by deficiencies in bone matrix material properties. However, cellular and molecular mechanisms leading to obesity-related bone fragility are not fully understood. Using male mouse models of obesity, we discovered TGF-β signaling plays a critical role in mediating the effects of obesity on bone. High-carbohydrate and high-fat diets increase TGF-β signaling in osteocytes, which impairs their mitochondrial function, increases cellular senescence, and compromises perilacunar/canalicular remodeling and bone quality. By specifically inhibiting TGF-β signaling in mouse osteocytes, some of the negative effects of high-fat and high-carbohydrate diets on bones, including the lacunocanalicular network, perilacunar/canalicular remodeling, senescence, and mechanical properties such as yield stress, were mitigated. DMP1-Cre–mediated deletion of TGF-β receptor II also blunted adverse effects of high-fat and high-carbohydrate diets on energy balance and metabolism. These findings suggest osteocytes are key in controlling bone quality in response to high-fat and high-carbohydrate diets. Calibrating osteocyte function could mitigate bone fragility associated with metabolic diseases while reestablishing energy balance.

## Abstract 2

Osteocytic TGFβ signaling, in part, relays the negative effects of high-fat and high-carbohydrate diets on bone mechanics and energy balance.

## Introduction

Although obesity and skeletal disease have continued to rise globally, poor skeletal health in obesity is often overlooked. Obesity was long thought to have an anabolic effect on bones because of increased weight-bearing (1, 2). Since people with obesity exhibit normal bone mineral density, they are not clinically diagnosed as individuals at high risk for fractures (3). However, many studies recently have shown that, despite having intact or high bone mineral density, people with obesity are prone to fractures (3). These fractures are due to bone quality deficits, a term encompassing many bone mineral density–independent features, including geometry, trabecular microarchitecture, and material properties (4–6). Although several aspects of bone quality are perturbed by obesity, underlying cellular and molecular mechanisms by which obesity compromises bone quality to cause fragility remain unclear.

Obesity develops from excess caloric intake relative to expenditure, from elevated fats, carbohydrates, or other nutrient sources (7–10). The most well-established mouse model for obesity induction involves feeding a high-fat diet (HFD) (Research Diet D12492) (11, 12). As a control for HFD, a diet low in fat-derived calories, i.e., low-fat diet (LFD) (Research Diet D12450J), is most widely used. This diet contains high carbohydrates to replace fat and support the caloric burden needed for energy production. Although the effects of this low-fat/high-carbohydrate diet on systemic metabolic regulation are similar to the regular animal chow diet (RD) (13, 14), controlled studies comparing the effect of a low-fat/high-carbohydrate diet and RD on bone quality are lacking. A recent study showed that high-carbohydrate diet affects bone remodeling and induces femoral and alveolar bone loss (15). A high-carbohydrate diet could adversely affect bone stiffness (15). Thus, a more detailed assessment of the effect of nutrient excess on bone is warranted.

Clinical and experimental studies place TGF-β at the intersection of obesity and bone fragility. Circulating TGF-β levels are markedly elevated in obese humans and murine models, including *ob/ob* mice and chronically HFD-fed mice (16–18). Elevated TGF-β/Smad 3 signaling is also associated with systemic insulin resistance and hepatic steatosis (17). Systemic neutralization or inhibition of TGF-β in HFD-induced obese mice ameliorates these phenotypes, suggesting TGF-β signaling contributes to the progression of obesity and obesity-related comorbidities (19–21). At the cellular level, TGF-β accelerates the acquisition of senescence-associated features in various cell types, including fibroblasts, epithelial cells, and cancer cells (22–26). Similarly, obesity and dyslipidemia can increase senescence markers, such as senescence-associated β-galactosidase activity and p16, p19, p21, and p53 gene expression in many cell types, including cells of adipose tissue, aorta, pancreas, liver, and endothelium (27–32). However, the contribution of TGF-β signaling to cellular senescence in obesity or to the obesity-associated loss of bone material properties has yet to be ascertained.

TGF-β signaling controls bone quality (33–37). Either excessive or insufficient TGF-β signaling can exacerbate bone fragility, and several mouse models further demonstrate the requirement for homeostatic levels of TGF-β signaling in bone (33–37). Osteocytes, which constitute the majority of bone cells, were shown to rely heavily on TGF-β signaling to maintain bone matrix material properties through perilacunar/canalicular remodeling (PLR), and repression of osteocytic TGF-β signaling suppressed PLR and severely compromised bone quality (38, 39). In obesity, the extent to which loss of bone quality occurs through a TGF-β– or osteocyte-dependent PLR mechanism remains undetermined.

We examined the impact of an HFD, a low-fat/high-carbohydrate diet, and an RD on TGF-β signaling in osteocytes. Our studies highlight that high-fat and low-fat/high-carbohydrate diets compromise bone quality. High-fat and high-carbohydrate diets increase PLR through osteocyte-intrinsic regulation of enzymes implicated in PLR, compromise lacunocanalicular network integrity and mitochondrial function, and induce cellular senescence. Using a mouse model targeted to delete TGF-β receptor II (TβRII) in late osteoblasts and osteocytes, we also demonstrate that suppression of TGF-β signaling moderates the effect of nutrient excess on cellular senescence, PLR, and bone fragility. Additionally, mice lacking a functional TβRII in late osteoblasts and osteocytes developed a marked reduction in weight gain and enhanced glucose tolerance and insulin sensitivity on high-fat and low-fat/high-carbohydrate diets. This study identifies a role for osteocytic TGF-β signaling in bone fragility and energy imbalance observed in obesity.

## Results

### High-fat and high-carbohydrate diets compromise bone quality.

Most animal studies that mimic the polygenic nature of human obesity use chronic HFD administration to induce obesity. Both RD and purified LFD are common choices for a corresponding control diet that is isoenergetic and relatively low in fat content. However, these control diets possess substantial nutritional differences that are often overlooked, especially in carbohydrate content and source (Table 1). Relative to RD, the recommended control LFD is a high-carbohydrate diet (55% carbohydrates in chow vs. 70% in LFD) enriched in easily metabolizable sources, such as sucrose (0.7% in chow vs. 7% in LFD). Furthermore, the content and source of fiber and lipids differ substantially. Recognizing that these differences in nutrient sources may affect bone, we compared the effects of HFD-induced obesity to RD and the purified low-fat/high-carbohydrate diet (HCD).

**Figure 1:**
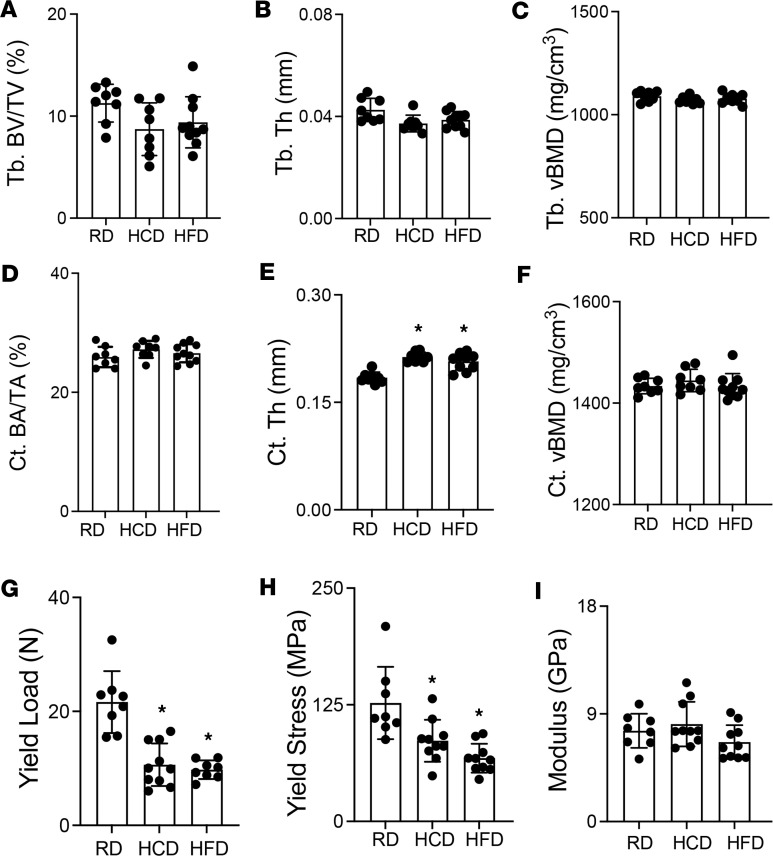
High-fat diet and its suggested control low-fat, high-carbohydrate diet impair bone material properties without affecting bone mineral density. μCT analysis on femurs of 30-week-old control male mice on standard chow diet (RD), low-fat/high-carbohydrate diet (HCD), and high-fat diet (HFD) shows changes in trabecular bone volume fraction (Tb. BV/TV) (**A**), thickness (Tb. Th.) (**B**), and volumetric bone mineral density (Tb. tBMD) (**C**) and cortical bone area fraction (Ct. BA/TA) (**D**), thickness (Ct. Th.) (**E**), and volumetric bone mineral density (Ct.tBMD) (**F**) in response to diets (*N* = 8–10). Flexural testing of femurs from RD-, HCD-, and HFD-fed mice shows a decline in bone material properties of yield load (**G**), yield stress (**H**), and bending modulus (**I**) (*N* = 8–10 mice/group). Data for **A**–**I** are presented as mean ± SD, and statistically significant differences, *P < 0.05, were determined with 1-way ANOVA and Newman-Keuls multiple post hoc test correction for the indicated group comparisons. Note: data from RD, HCD, and HFD control mice in Figure 1 are replicated in Figure 8.

As shown in Figure 1, A–C, compared to RD, HCD and HFD had no marked effects on trabecular bone volume fraction, number, and bone mineral density in wild-type male mice. A subtle yet substantial increase in trabecular thickness was observed in the bones of HCD- and HFD-fed wild-type mice (Table 2). Cortical bone volume fraction and bone mineral density were unaffected by diet, but a subtle yet significant increase in cortical thickness was observed in HCD- and HFD-fed mouse bones compared with RD (Figure 1, D–F, and Table 2). At the whole bone level, HFD-fed mouse bone showed the anticipated decline in mechanical properties relative to RD, with an approximately 50% reduction in femoral yield load, yield stress, and ultimate stress (Figure 1, G–I, and Table 3) (40–43). Stiffness, fracture load, post-yield displacement, and elastic modulus were unchanged by HFD. Surprisingly, HCD-fed mouse bone also demonstrated considerable declines in yield load, ultimate load, post-yield displacement, yield stress, and ultimate stress relative to RD controls (Figure 1, G–I, and Table 3). These findings are remarkable in that they not only recapitulate the detrimental effects of the HFD on bone quality (4, 42) but also reveal the substantial adverse effects of the HCD on bone mechanical properties compared with the RD. Thus, careful consideration is needed to select the appropriate control diet while studying skeletal manifestations of metabolic disease.

### High-fat and high-carbohydrate diets increase osteocyte intrinsic TGF-β signaling.

**Figure 2:**
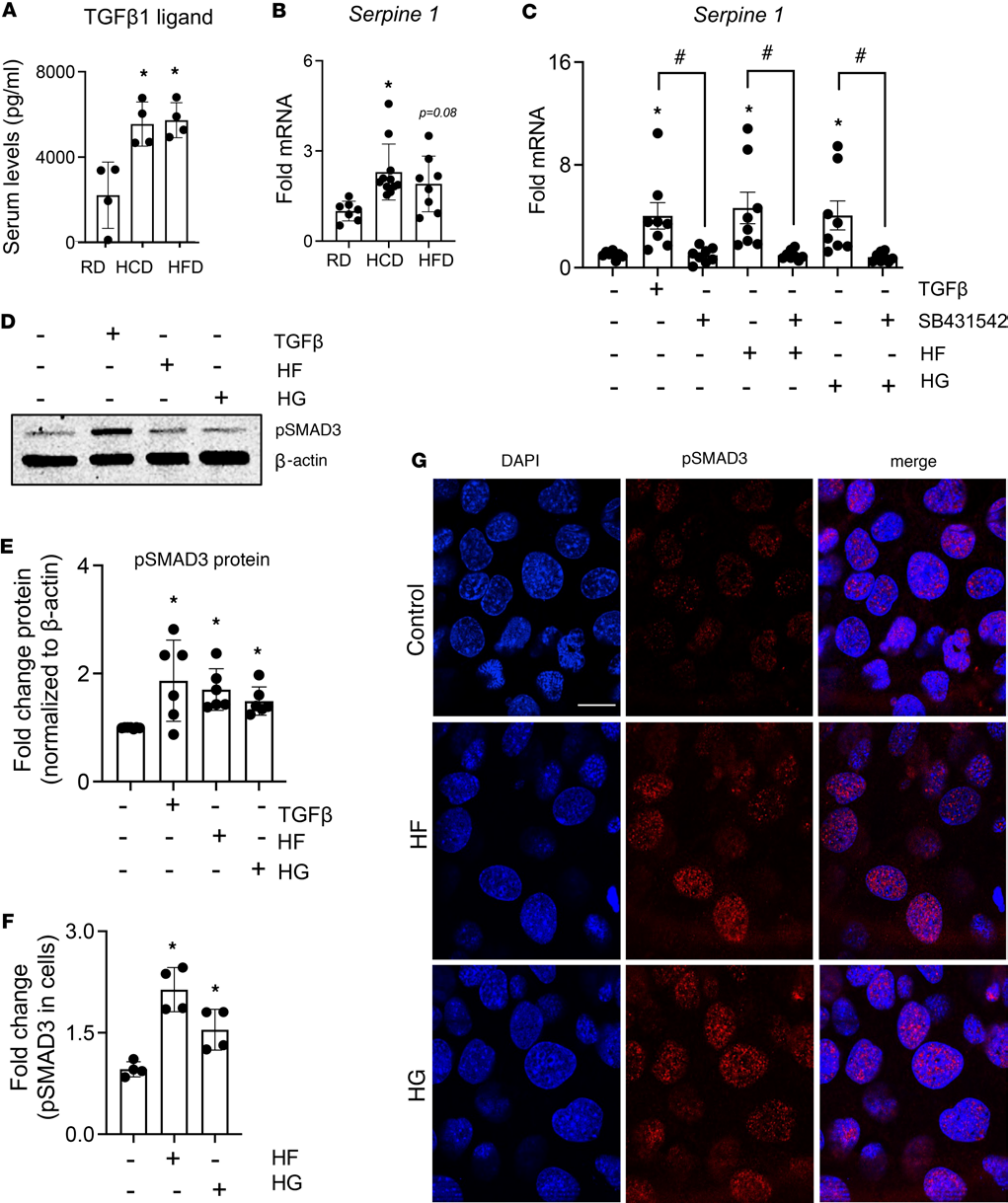
High-carbohydrate and -fat diets increase cell-intrinsic TGF-β signaling in osteocytes. TGF-β1 levels in serum (**A**) (*N* = 4 mice/group) and mRNA levels of TGF-β–responsive gene, Serpine 1, in cortical bones (**B**) (*N* = 7–11 mice/group) of 30-week-old mice fed a standard chow diet (RD), low-fat/high-carbohydrate diet (HCD), or high-fat diet (HFD) were determined. For **A** and **B**, *P < 0.05 different from the RD-fed mice, and data are presented as mean ± SD. Differentiated OCY454 cells were treated with high fatty acids (HF, palmitate 100 μm, oleate 200 μm, linoleate 200 μm), high glucose (HG, 25 mM), TGF-β (5 ng/mL), or TGF-β receptor I kinase inhibitor, SB431542 (10 μM), for 72 hours for real-time quantitative PCR (qRT-PCR) (**C**), immunoblotting (**D** and **E**), and immunofluorescence (**F** and **G**). Changes in Serpine 1 mRNA (**C**) in the presence of HF, HG, and TGF-β (*N* = 8 biological replicates/group, compiled from 3 independent experiments, presented as mean ± SD). Induction of phosphorylated Smad 3 (**D** and **E**) with HF, HG, and TGF-β treatments was detected (*N* = 6 biological replicates/group, compiled from 3 independent experiments with *N* = 2 biological replicates/group/experiment, data are presented as mean ± SD). Immunofluorescence for increased Smad 3 nuclear localization and activation (**F** and **G**) with HF and HG treatments (*N* = 3 biological replicates/condition, 3 regions of interest [ROIs]/mouse, and data are shown as mean ± SD, and reproduced across 2 independent experiments). Scale bar is 50 µm. *P < 0.05 different from the control (untreated group), and ^#^*P* < 0.05 denotes the difference in the presence of SB431542 for each of the treatments: TGF-β, HF, or HG. Statistical differences were determined with 1-way ANOVA and Newman-Keuls multiple post hoc correction (**A**–**C**, **E**, and **F**).

Serum TGF-β levels are systemically increased with obesity in humans and with HFD in mice (16, 19). We also detected increased TGF-β1 ligand levels in the serum of HFD- and HCD-fed mice (Figure 2A). To evaluate the effect of HFD or HCD on TGF-β signaling in bone, we measured mRNA expression of a downstream target of the TGF-β signaling pathway, *Serpine 1*, and of *TβRII*. Compared with RD, HCD increased *Serpine 1* (4-fold) and *TβRII* (>6-fold) mRNA in cortical bone (*P* < 0.01, Figure 2B and Supplemental Figure 5, A and I; supplemental material available online with this article; https://doi.org/10.1172/jci.insight.175103DS1). Bone from HFD-fed mice had unchanged *TβRII* mRNA levels but showed a trend toward increased *Serpine 1* mRNA (4-fold, *P* < 0.1, Figure 2B). To assess osteocyte-intrinsic effects of nutrient excess on TGF-β signaling, we exposed OCY454 osteocyte-like cells to high–fatty acid (HF) or high-glucose (HG) culture conditions for 72 hours to model chronic HFD and HCD feeding. Like TGF-β, HF and HG treatments induced *Serpine 1* mRNA levels (~5-fold). Furthermore, the TGF-β receptor I kinase inhibitor (SB431542) mitigated both the HF- and HG-mediated induction of *Serpine 1* (Figure 2C). HF increased Smad 3 phosphorylation (Figure 2, D and E), and HF and HG increased nuclear localization of phosphorylated Smad 3 (Figure 2, F and G). Together, these results verify the ability of elevated fatty acid and glucose to stimulate osteocyte-intrinsic TGF-β signaling.

### High-fat and high-carbohydrate conditions reduce mitochondrial function.

**Figure 3:**
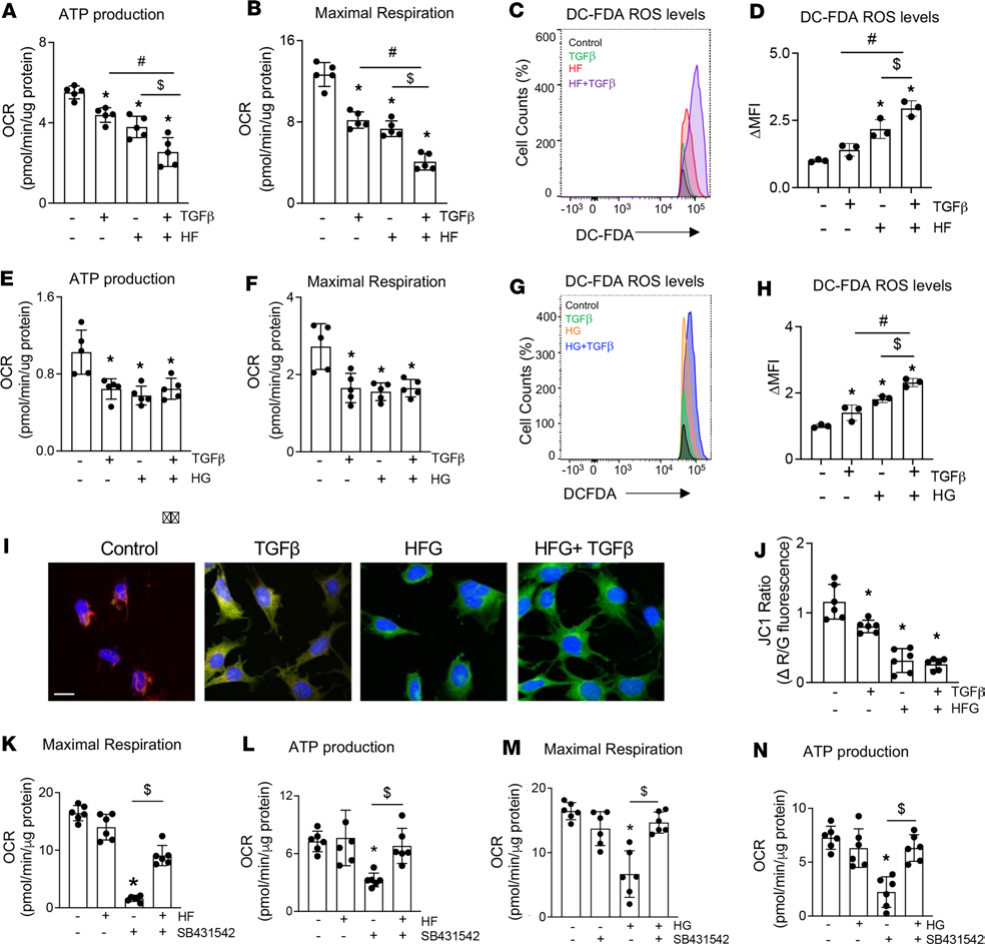
Hyperglycemia and hyperlipidemia affect osteocyte intrinsic cellular metabolism. Undifferentiated OCY454 cells were exposed to hyperlipidemia (HF, palmitate 100 μm, oleate 200 μm, linoleate-200 μm) or hyperglycemia (HG, glucose 25 mM) in the presence or absence of TGF-β (5 ng/mL) or TGF-β receptor I kinase inhibitor, SB431542 (10 μM), for 24 hours. In response to HF, HG, and TGF-β treatment, changes in oxygen consumption rate (OCR) parameters, namely, ATP production (**A** and **E**) and maximum respiration (**B** and **F**), were measured. Changes in intracellular ROS with HF (**C** and **D**) and HG (**G** and **H**) treatment determined with DCFDA stain have been quantified as mean fluorescence intensity (MFI) (*N* = 3 technical replicates/condition). Changes in mitochondrial membrane potential (**I** and **J**) with JC1 dye with TGF-β, HFG (palmitate, oleate, linoleate, glucose), or a combination of both treatments were quantified as red-to-green fluorescence intensity ratios (R/G) from sum projections, then normalized to the control group (for **I**, scale bar is 5 μm; for **J**, *N* = 3 technical replicates/condition). Changes in OCR parameters, namely, maximum respiration (**K** and **M**) and ATP production (**L** and **N**) in HF-, HG-, and SB431542-treated OCY454 cells have been shown. (*N* = 5 technical replicates/condition.) For **A**–**N**, data are presented as mean ± SD and were reproduced across 3 independent experiments. *P < 0.05 different from the untreated group, ^#^*P* < 0.05 different from the TGF-β–treated group, ^$^*P* < 0.05 different from HF- or HG-treated groups, and differences were calculated with 2-way ANOVA and Newman-Keuls multiple post hoc correction. Statistical interactions are provided in Supplemental Tables 3 and 4.

Long-term HFD consumption suppresses expression of genes regulating mitochondrial biogenesis and activity in several tissues (44–48). Similarly, excess dietary carbohydrates induce oxidative stress and compromise mitochondrial integrity in muscle cells (49). To elucidate mechanisms responsible for the effects of HCD and HFD on bone, we examined the osteocyte-intrinsic impact of HF and HG conditions on mitochondrial function in OCY454 osteocyte-like cells. Moreover, since TGF-β is a potent modulator of mitochondrial function (50), we examined the extent to which osteocytic TGF-β signaling is required for these effects. For analysis of real-time mitochondrial respiration in osteocytes, we utilized a Seahorse extracellular flux analyzer that measures oxygen consumption rates (OCRs) before and after the addition of compounds that target complexes I and III of the respiratory chain, antagonize the ATP synthase, or uncouple mitochondrial oxidative phosphorylation (OXPHOS) to enable analysis of numerous mitochondrial parameters. Compared with osteocytes incubated in control conditions, exposure to exogenous TGF-β ligand lowered OCR, substantially reducing ATP production and maximal respiratory capacity (Figure 3, A, B, E, and F). Like TGF-β, HF and HG suppressed mitochondrial OCR. While the combination of TGF-β and HF further suppressed OCR (Figure 3, A and B, and Supplemental Figure 1, A–F), the effect of combined TGF-β and HG on OCR did not differ from either stimulus alone (Figure 3, E and F; Supplemental Figure 1, G–K; and Supplemental Tables 3 and 4).

Furthermore, TGF-β, HF, and HG increased cellular ROS (Figure 3, C, D, G, and H), and TGF-β augmented HF-mediated, but not HG-mediated, increases in cellular ROS (Supplemental Tables 3 and 4). Along with reducing mitochondrial activity in osteocytes, HF, HG, and TGF-β caused mitochondrial membrane depolarization (Figure 3, I and J). This suggests increased osteocyte exposure to free fatty acids, glucose, or TGF-β leads to reduced mitochondrial activity and increased cellular ROS.

Since HF and HG regulate mitochondrial function, which is consistent with the effect of TGF-β, we tested the causal role of TGF-β signaling in HF and HG’s effect on osteocyte mitochondrial function using the TβRI antagonist SB431542. In basal conditions, SB431542 did not affect the osteocyte mitochondrial OCR parameters; however, SB431542 alleviated both HF- and HG-dependent repression of mitochondrial function (Figure 3, K–N, and Supplemental Figure 3, A–H).

In contrast, TGF-β stimulates osteocytic extracellular acidification rates (ECAR), as reflected by the increased glycolysis and glycolytic capacity, which differs from the glycolysis-suppressing effect of HF and HG (Supplemental Figure 2, A–J, and Supplemental Tables 3 and 4). Inhibition of TGF-β signaling by SB431542 suppressed glycolytic function in osteocytes (Supplemental Figure 3, I–P). Surprisingly, SB431542 could not alleviate the repression of cellular glycolysis mediated by HF and HG (Supplemental Figure 3, I–P, and Supplemental Tables 3 and 4). Overall, these findings implicate a cell-intrinsic role for TGF-β signaling in the regulation of osteocyte mitochondrial function by both HF and HG, through mechanisms that appear to be distinct. Furthermore, the HF and HG effects on glycolysis are independent of TGF-β signaling.

### Accelerated osteocyte senescence is induced by high-carbohydrate and high-fat diets.

**Figure 4:**
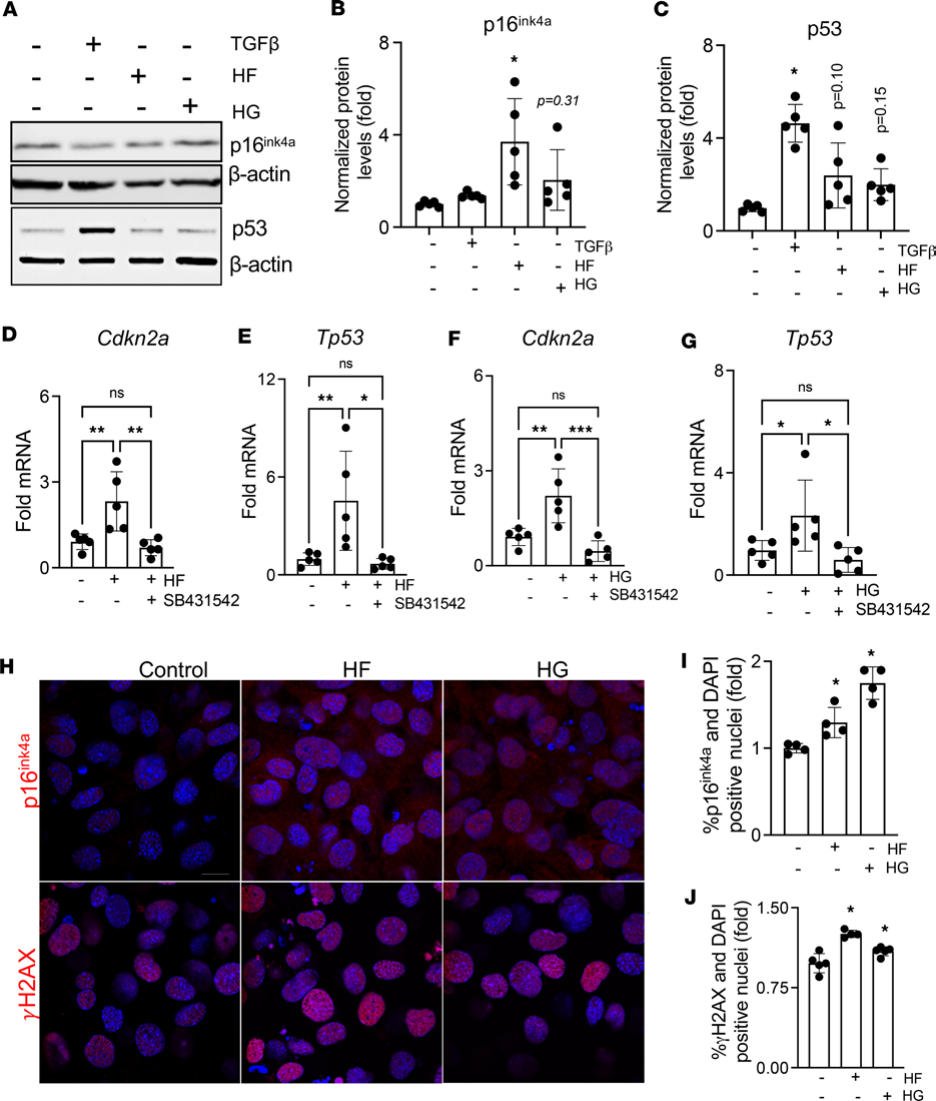
Hyperglycemia and hyperlipidemia promote cellular senescence through TGF-β signaling in osteocytes. Differentiated OCY454 cells were treated with high fatty acids (HF, palmitate 100 μm, oleate 200 μm, linoleate 200 μm), high glucose (HG, 25 mM), TGF-β (5 ng/mL), or TGF-β receptor I kinase inhibitor, SB431542 (10 μM), for 72 hours. Immunoblotting shows induction in p16^ink4a^ and p53 in the presence of HF, HG, or TGF-β (**A**–**C**) (*N* = 2 biological replicates/group, and data compiled from 3 independent experiments). Quantitative real-time PCR (qRT-PCR) shows increased mRNA levels of senescence markers, Cdkn2a (**D** and **F**) and Tp53 (p53) (**E** and **G**) with HF and HG with or without SB431542 treatment. Immunofluorescence shows an increased percentage of DAPI-positive osteocytes that also stained positive for p16^ink4a^ (**H** and **I**) and γH2AX (**H** and **J**) (*N* = 3 technical replicates/group repeated in 3 independent experiments). Scale bar is 50 µm. Data for **A**–**J** are shown as mean ± SD and *P < 0.05, **P < 0.01, ***P < 0.005; differences were calculated with 1-way ANOVA and Newman-Keuls multiple post hoc correction. Statistical interactions are provided in Supplemental Table 5.

Since deteriorated mitochondrial OXPHOS is involved in the early stages of cellular senescence (51), we examined if high-carbohydrate and high-fat conditions also increase osteocyte senescence. First, we investigated whether HF and HG induced cellular senescence in differentiated OCY454 cells by examining senescence markers *Cdkn2a* (p16^ink^), *Tp53* (p53), p21^cip^, and γH2A.X. Western blot showed considerable induction of senescent marker protein levels by TGF-β, HF, and HG in cultured osteocytes (Figure 4, A–C). The increased protein levels of p16^ink4a^ and p53 (a trend) in osteocytes by HF and HG, respectively, were corroborated with both HF and HG at the mRNA level. Additionally, the HF- and HG-induced expression of *Cdkn2a* (p16^ink^) and *Tp53* (p53) was blocked by the TβRI inhibitor, SB431542 (Figure 4, D–G), which alone had no effect (Supplemental Figure 4). Evidence of HF- and HG-induced senescence was strengthened by our detection of nuclear expression of the DNA damage marker γH2A.X and p16^ink4a^ using immunofluorescence. Both γH2A.X level and p16^ink^-positive particles remarkably increased after HF and HG exposure (Figure 4, H–J). These findings suggested that HF and HG exposure could induce classical senescence marker gene expression in osteocytes and that blocking TGF-β signaling could mitigate these effects.

**Figure 5:**
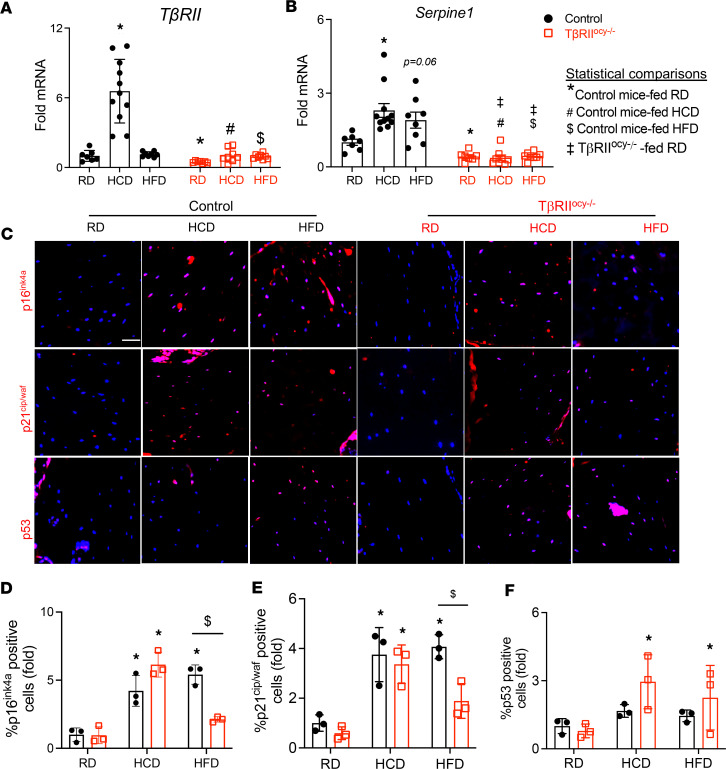
High-fat diet–induced osteocyte senescence is TGF-β dependent. Quantitative real-time PCR (qRT-PCR) of cortical bone-derived RNA from 30-week-old control and TβRII^ocy–/–^ male mice fed a standard chow diet (RD), low-fat/high-carbohydrate diet (HCD), or high-fat diet (HFD) used for in vivo assessment of TβRII and Serpine 1 expression in osteocytes (**A** and **B**) (*N* = 7–11 mice/group, mean ± SD). Immunohistochemistry (IHC) for p16^ink4a^, p21^cip/waf^, and p53 was conducted on femoral cortical bone sections. Representative images (**C**) of IHC and the percentage of DAPI-stained osteocytes that also stained for p16^ink4a^ (**D**, top), p21^cip/waf^ (**E**, middle), and p53 (**F**, bottom) were quantified and expressed as fold-change, relative to RD-fed control mice (*N* = 3 mice/group and 4 regions of interest [ROI]/mouse were collected, mean ± SD, the scale bar is 50 μm). *P < 0.05 different from RD-fed control mice, ^#^*P* < 0.05 different from HCD-fed control mice, ^$^*P* < 0.05 different from HFD-fed control mice, ^‡^*P* < 0.05 different from RD-fed TβRII^ocy–/–^ mice, as calculated from the 2-way ANOVA and Newman-Keuls multiple post hoc correction. Statistical interactions are provided in Supplemental Table 6.

To determine if HFD- and HCD-induced osteocyte senescence can be relieved by TGF-β inhibition in vivo, we used immunohistochemistry to examine expression of senescence markers in bone sections from TβRII^ocy–/–^ mice, which show marked reduction in TGF-β signaling (Figure 5, A and B), and their control littermates. Both control and TβRII^ocy–/–^ mice on RD showed minimal expression of senescence markers p16^ink^, p21^cip/waf^, and p53. HCD- and HFD-fed control mice showed high levels of p16^ink4a^ and p21^cip/waf^ (~5-fold induction, Figure 5, C–E, and Supplemental Table 6), with no apparent changes in p53 (Figure 5, C and F, and Supplemental Table 6). While osteocytic TGF-β inhibition did not mitigate the effects of HCD on senescent markers, the HFD-fed TβRII^ocy–/–^ mice had considerably fewer p16^ink^- and p21^cip/waf^-positive osteocytes than HFD-fed genotype controls (Figure 5, C–E, and Supplemental Table 6). This suggests that HFD-mediated senescence in osteocytes relies in part on TGF-β signaling. Together, these experiments indicate that senescent osteocytes accumulate with high-fat and -carbohydrate diets in bone and that TGF-β signaling is partially involved in this effect.

### Ablation of osteocytic TGF-β signaling mitigates the induction in PLR caused by high-fat and high-carbohydrate diets.

**Figure 6:**
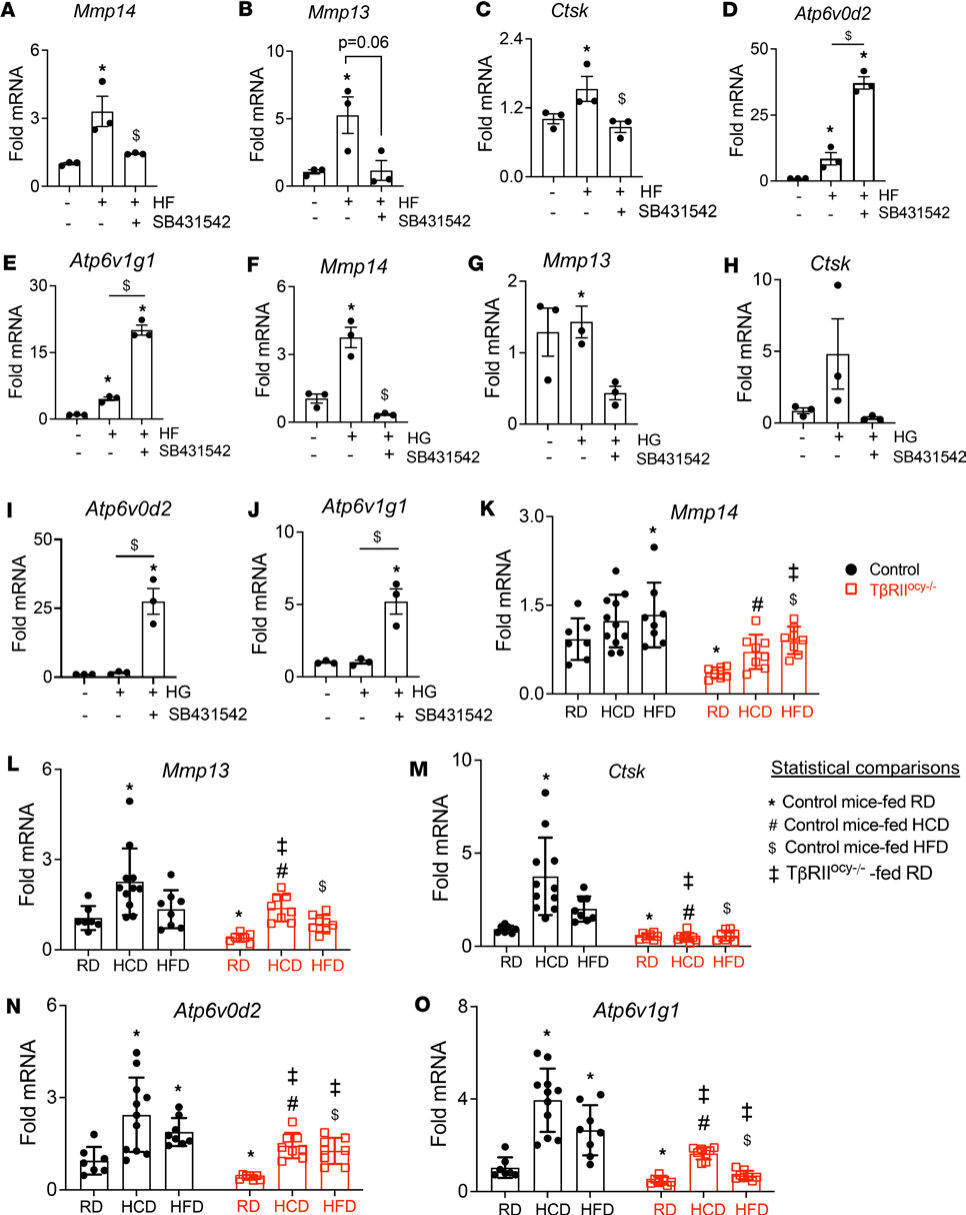
High-carbohydrate and high-fat diet–induced osteocytic perilacunar/canalicular remodeling gene expression is partially TGF-β dependent. Quantitative real-time PCR (qRT-PCR) shows differential regulation of genes implicated in PLR, *Mmp13*, *Mmp14*, *Ctsk*, *Atp6v0d2*, and *Atp6v1g1*, when differentiated OCY454 cells were subjected to hyperlipidemia (HF, **A**–**E**) or hyperglycemia (HG, **F**–**J**) in the presence or absence of SB431542 (10 μM) for 72 hours (*N* = 3 biological replicates/group, replicated in 3 independent experiments, *P < 0.05 different from untreated group, ^$^*P* < 0.05 different from HF or HG treatment). mRNA levels of *Mmp13*, *Mmp14*, *Ctsk*, *Atp6v0d2*, and *Atp6v1g1*, in cortical bones of 30-week-old control and TβRII^ocy–/–^ mice fed a standard chow diet (RD), low-fat/high-carbohydrate diet (HCD), or high-fat diet (HFD) were quantified (**K**–**O**), (*N* = 7–11 mice/group). *P < 0.05 different from RD-fed control mice, ^#^*P* < 0.05 different from HCD-fed control mice, ^$^*P* < 0.05 different from HFD-fed control mice, ^‡^*P* < 0.05 different from RD-fed TβRII^ocy–/–^ mice; differences were calculated with 1-way (**A**–**J**) and 2-way (**K**–**O**) ANOVA, with Newman-Keuls multiple post hoc corrections, and data shown as mean ± SD (**A**–**O**). Statistical interactions are provided in Supplemental Table 6.

We showed that osteocyte-intrinsic TGF-β signaling is required for PLR (38, 39). Therefore, we sought to examine if HCD- and HFD-inducible TGF-β signaling in osteocytes stimulated PLR. Using OCY454 osteocyte-like cells that were differentiated for 3 days to mimic osteocytic gene expression (52), we examined the effects of exposure to high fatty acid or glucose for 72 hours on expression of genes implicated in PLR (38, 39), including *Mmp13*, *Mmp14*, and cathepsin K (*Ctsk*). We found that HF increased the levels of *Mmp13*, *Mmp14*, and *Ctsk* mRNA but that antagonism of TGF-β signaling (using TβRI inhibitor SB431542) relieved this coordinated induction in PLR genes (Figure 6, A–E). Similarly, HG substantially increased osteocytic Mmp14 and showed a trend of inducing Ctsk mRNA levels, while SB431542 mitigated HG-induced expression of these genes (Figure 6, F–J). Together, these results suggested that both HF and HG can increase mRNA levels of osteocytic genes required for PLR via a TGF-β–dependent mechanism.

To determine if osteocytic TGF-β signaling is required for the effects of HCD and HFD on bone, we used previously characterized TβRII^ocy–/–^ mice that have targeted ablation of osteocytic TβRII expression (38, 39) (Figure 5, A and B). We examined the effects of HCD, HFD, or standard chow feeding on the expression of genes implicated in PLR in the bones of control and TβRII^ocy–/–^ mice. We found that bones from the control mice fed HCD exhibited increased *Mmp13* (~2.5-fold) and *Ctsk* (~3.5-fold) mRNA levels. Bone from HFD-fed control mice, on the other hand, only showed significant elevation in the mRNA levels of *Mmp14* while showing a trend toward increased *Mmp13* and *Ctsk* levels (Figure 6, K–O).

In TβRII^ocy–/–^ mice fed RD, *Mmp13*, *Mmp14*, and *Ctsk* mRNA levels were reduced relative to littermate controls (Figure 6, K–M), which was in congruence with our previous findings (1, 2). Although both HCD and HFD induced *Mmp13* and *Mmp14* in TβRII^ocy–/–^ mice, their mRNA levels were identical to those in the RD-fed controls (Figure 6, K and L). On the other hand, effects of HCD and HFD on *Ctsk* mRNA were abolished in TβRII^ocy–/–^ mice (Figure 6M), indicating HCD and HFD need osteocytic TGF-β to increase *Ctsk* in osteocytes. ATPases implicated in PLR activity, *Atp6v1g1* and *Atp6v0d2*, were also induced by both HCD and HFD in control and TβRII^ocy–/–^ bone (Figure 6, N–O). Interestingly, in TβRII^ocy–/–^ mice, the HCD or HFD induction in *Atp6v1g1* and *Atp6v0d2* restored the expression to a homeostatic range similar to that observed in the RD-fed control mice. Not all genes implicated in PLR are affected by HCD and HFD. This is evident from relatively stable expression of *Mmp2* mRNA, which was only suppressed in response to ablated TGF-β signaling in TβRII^ocy–/–^ mice (Supplemental Figure 5, H and P).

**Figure 7:**
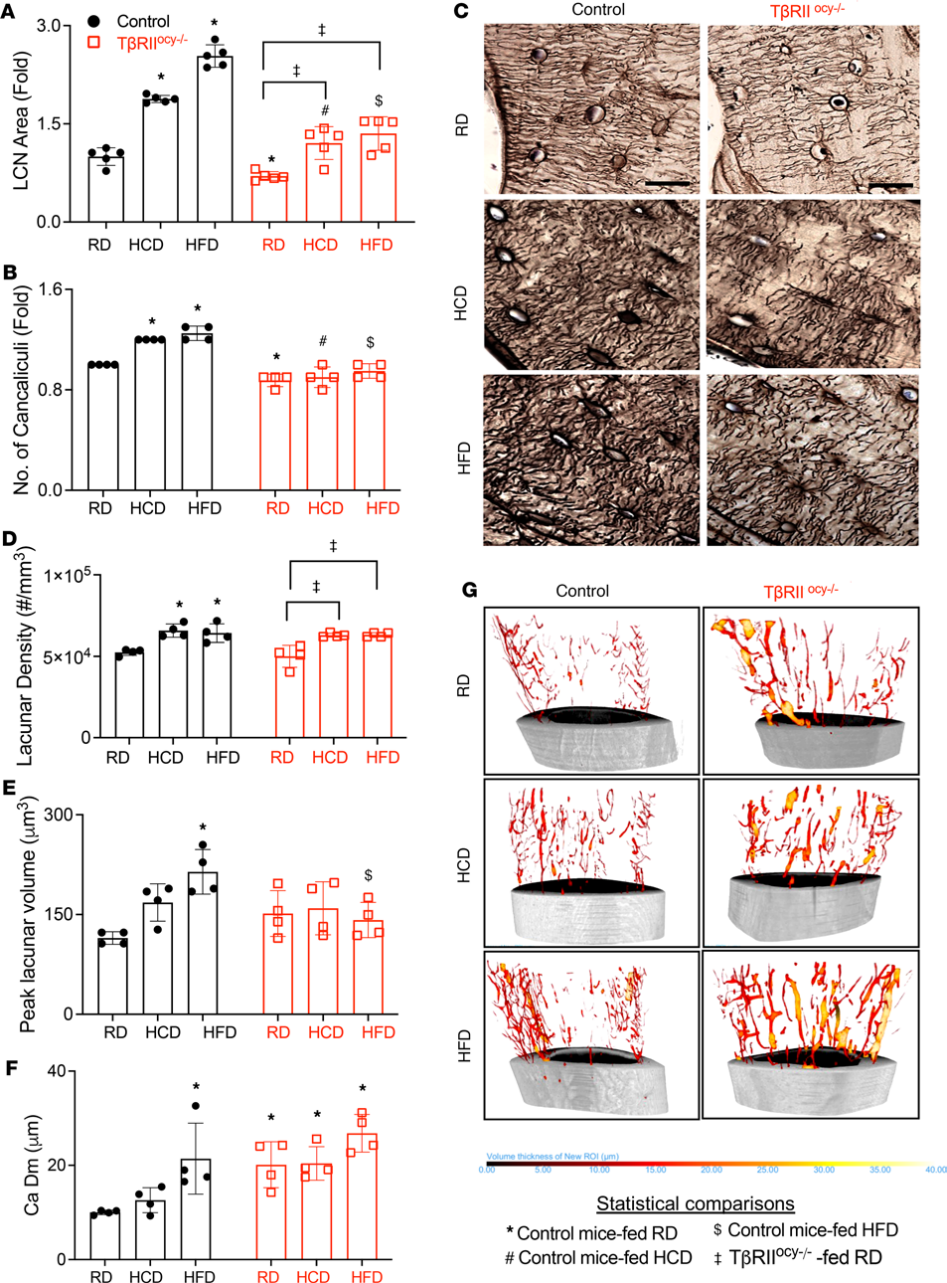
High-carbohydrate and -fat diets alter osteocyte lacunar volume and canalicular network, and ablation of TGF-β signaling lessens these dietary effects on osteocytes. Standard chow diet (RD), low-fat/high-carbohydrate diet (HCD), or high-fat diet (HFD) fed 30-week-old control and TβRII^ocy–/–^ mouse femurs were used for assessment of the osteocyte lacunocanalicular network (LCN) in femoral cortical bone with Ploton silver stain. Quantified LCN area (**A**), number of canaliculi (**B**), and representative images (**C**) have been shown (*N* = 5 mice/group with 4 ROI/mouse; data shown as mean ± SEM; scale bar = 20 μm). SRμCT was used to determine osteocyte lacunar density (**D**), volume (**E**), and vascular canal diameter (**F** and **G**) in tibial cortical bones of RD-, HCD-, or HFD-fed 30-week-old control and TβRII^ocy–/–^ mice (*N* = 4 mice/group; data are shown as mean ± SD). *P < 0.05 different from RD-fed control mice, ^#^*P* < 0.05 different from HCD-fed control mice, ^$^*P* < 0.05 different from HFD-fed control mice, ^‡^*P* < 0.05 different from RD-fed TβRII^ocy–/–^ mice, as calculated from the 2-way ANOVA and Newman-Keuls multiple post hoc correction. Statistical interactions are provided in Supplemental Table 6.

Since deregulation of the osteocyte lacunocanalicular network (LCN) is also a hallmark of erratic PLR, we evaluated whether the diets adversely affect the LCN via TGF-β–dependent mechanisms. Two-dimensional histological analysis of the LCN with Ploton silver stain revealed that both HCD- and HFD-fed control mice exhibited dense canalicular networks, with differences attributed to augmented LCN area (~2-fold) and increased number of canaliculi per osteocyte (Figure 7, A–C). In RD-fed TβRII^ocy–/–^ mice, LCN area and canalicular number were reduced, as previously reported (Figure 7, A–C) (38, 39). HCD and HFD increased the LCN area in TβRII^ocy–/–^ bone such that their LCN phenotype resembled the RD-fed control mice (Figure 7, A–C), indicating a normalizing effect of TβRII ablation relative to diets. Canalicular number, however, remained low in TβRII^ocy–/–^ bone, and neither HCD nor HFD affected canalicular number (Figure 7, A–C).

Synchrotron radiation micro-computed tomography (SRμCT), which visualizes and quantifies osteocyte lacunae and vascular canals in a 3-dimensional space, revealed changes in osteocyte lacunar features with diet (Figure 7, D and E). Particularly, lacunar density was higher in bone from HCD- and HFD-fed mice of both genotypes. Consistent with prior reports, no genotype-dependent differences in lacunar density were observed (38, 39). Peak lacunar volume was appreciably increased by HFD in control bone but reduced by HFD in TβRII^ocy–/–^ bone (Figure 7E). Further, we investigated the effect of diet and genotype on the density and diameter of bone vascular canals. The density of vascular canals was unaffected by diet or genotype (Supplemental Figure 5, Q and R). However, bone vascular canal diameter was increased by HFD in control mice (Figure 7, F and G). TβRII^ocy–/–^ mice on RD exhibited higher bone vascular canal diameter compared with RD control mice. HCD did not impact bone vascular canal diameter or density in either genotype (Figure 7, F and G, and Supplemental Figure 5, Q and R). Collectively, our results examining hallmarks of PLR suggest that high-carbohydrate and high-fat diets increase osteocytic PLR, altering expression of selective genes implicated in PLR and LCN architecture, and that some of these effects depend on osteocytic TGF-β signaling.

### Disrupting osteocytic TGF-β signaling limits HFD-dependent decline in bone yield stress.

**Figure 8:**
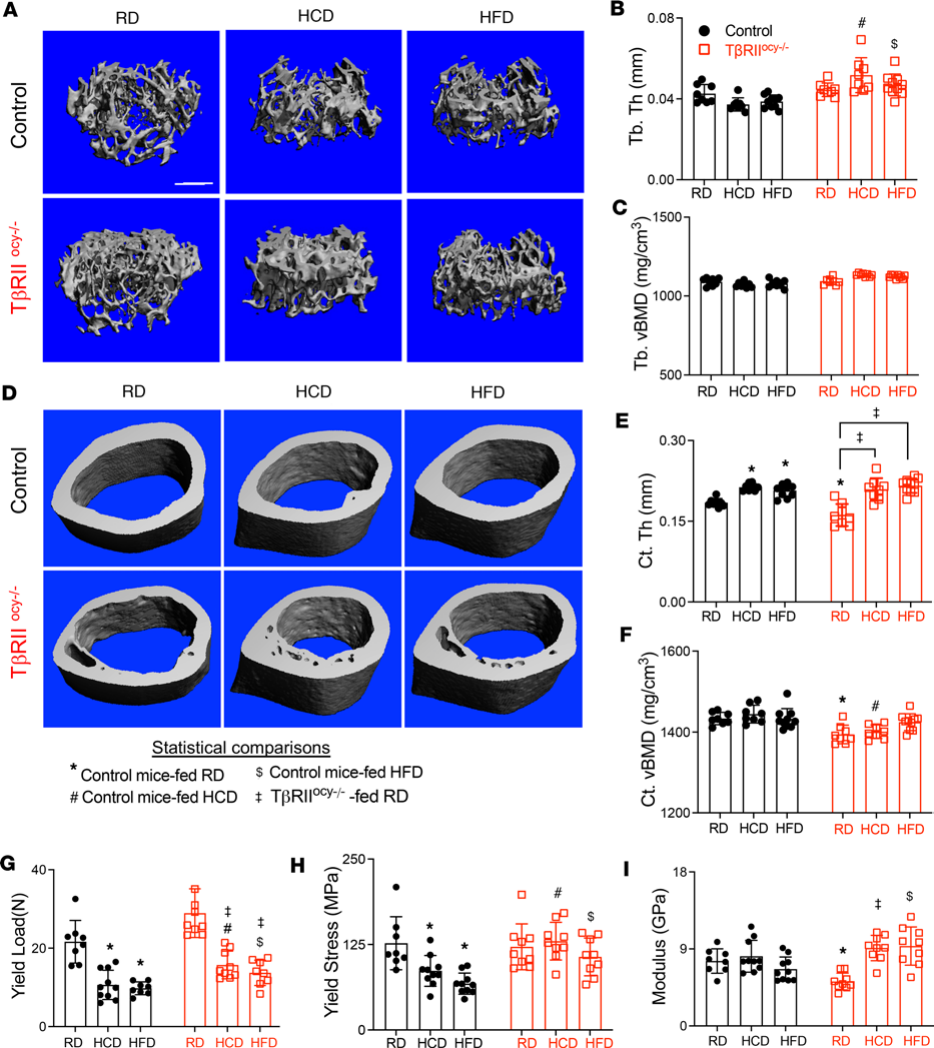
Disruption of TGF-β signaling in osteocytes prevents high-carbohydrate and high-fat diet–induced bone fragility. μCT analysis of femoral bones from 30-week-old male control and TβRII^ocy–/–^ mice fed RD, HCD, or HFD shows changes in trabecular and cortical bone parameters. Representative μCT images (**A**–**D**, scale bar 100 μm) and analysis of trabecular and cortical bone parameters, namely, trabecular bone thickness (Tb.Th) (**B**), trabecular bone mineral density (Tb. tBMD) (**C**), cortical thickness (Ct. Th) (**E**), and cortical bone mineral density (Ct. tBMD) (**F**) are shown (*N* = 8–10 mice/group). Flexural strength tests of bones of control and TβRII^ocy–/–^ mice fed RD, HCD, or HFD reveal effects of diet on bone material properties, including yield load, yield stress, and elastic modulus (**G**–**I**) (*N* = 8–10 mice/group). For **A**–**I**, data are shown as mean ± SD; *P < 0.05 different from control mice fed RD, ^#^*P* < 0.05 different from control mice fed LFD, ^$^*P* < 0.05 different from control mice fed HFD, and ^‡^*P* < 0.05 different from TβRII^ocy–/–^ mice fed RD; statistical differences were calculated using 2-way ANOVA and Newman-Keuls multiple post hoc corrections. Statistical interactions are provided in Supplemental Tables 7 and 8. Note: data on RD, HCD, and HFD control mice are replicated in Figure 1.

Since high-carbohydrate and high-fat diets increase osteocyte-intrinsic TGF-β signaling, and since both diets can affect bone mass and quality, we investigated whether diet-induced bone fragility can be mitigated by inhibition of osteocytic TGF-β signaling. The trabecular and cortical bone phenotype of TβRII^ocy–/–^ mice on RD is consistent with our prior studies (38, 39). Specifically, relative to RD genotype controls, TβRII^ocy–/–^ mice on RD exhibited a high trabecular bone volume phenotype due to increased trabecular number (Figure 8, A–C, and Table 4). Cortical bone volume was unaffected in TβRII^ocy–/–^ mice despite a subtle decrease in cortical thickness (Figure 8, D and E, and Table 4). Cortical bone volumetric bone mineral density was reduced in the TβRII^ocy–/–^ mice on RD, relative to RD genotype controls.

Like the control mice, neither HCD nor HFD affected the trabecular bone volume of TβRII^ocy–/–^ mice, and these mice continued to retain high trabecular bone volume. Surprisingly, the cortical bone volume that remained unaltered by diets in the control mice was increased in the HCD- and HFD-fed TβRII^ocy–/–^ mice. This increased cortical bone volume was accompanied by increased cortical thickness, and similar to the control mice, TβRII^ocy–/–^ mice on HCD and HFD exhibited increased cortical thickness (Table 4 and Figure 8, D–F). Last, HCD- and HFD-fed TβRII^ocy–/–^ mice showed reduced and intact cortical bone mineral density, respectively, relative to RD TβRII^ocy–/–^ mice.

Next, we directly examined the effects of diets and osteocytic TβRII ablation on bone mechanical behavior in 3-point bending tests of femora. TβRII^ocy–/–^ mice on standard chow showed reduced elastic modulus and yield stress. Stiffness, yield load, fracture force, and work to fracture did not differ between the 7.5-month-old control and TβRII^ocy–/–^ mice on RD. Relative to RD, bone from control mice fed HCD and HFD showed an approximately 50% reduction in yield load and yield stress (Figure 8, G and H). In contrast, TβRII^ocy–/–^ mice fed HCD and HFD showed increased stiffness, elastic modulus, yield stress, and ultimate stress relative to genotype controls (Table 5). This suggests that TβRII^ocy–/–^ mouse bones are resistant to the HFD- or HCD-dependent decline in yield stress. Not all the effects of HCD and HFD on bone mechanical properties are mitigated in TβRII^ocy–/–^ mice. For example, the elastic modulus is elevated by both diets in TβRII^ocy–/–^ bone (Figure 8I). Nonetheless, osteocytic deficiency in TGF-β signaling attenuates some of deleterious effects of high-carbohydrate and high-fat diets on bone.

### Disrupting osteocytic TGF-β signaling blunts the effects of HFD on energy metabolism.

Systemic inhibition of TGF-β signaling in Smad 3–deficient mice or using an anti–TGF-β1 antibody has been reported to mitigate adverse effects of obesity and diabetes on energy metabolism (19). However, contributing cellular mechanisms remain unclear. Given the ability of mice with reduced osteocytic TGF-β signaling to counteract the impact of HFD on bone and the pivotal role of bone-derived factors in energy metabolism (53–55), we hypothesized that reduced osteocytic TGF-β signaling could ameliorate deleterious effects of HFD on energy metabolism.

**Figure 9:**
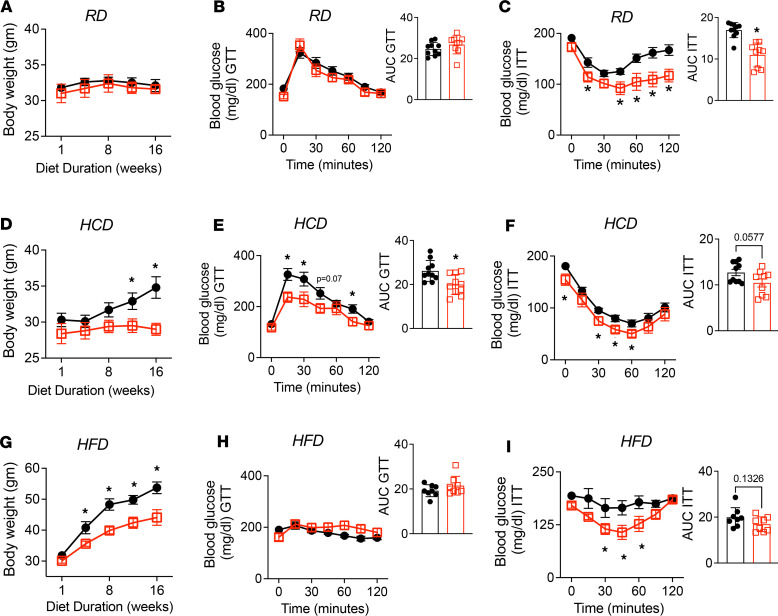
TβRII^ocy–/–^ mice are resistant to high-carbohydrate and high-fat diet–induced deregulation of energy metabolism. Body weight of male control and TβRII^ocy–/–^ mice fed standard chow diet (RD, **A**), low-fat/high-carbohydrate diet (HCD, **D**), or high-fat diet (HFD, **G**) is shown. Intraperitoneal glucose (GTT) and insulin tolerance tests (ITT) were performed at the end of diet in male control and TβRII^ocy–/–^ mice fed RD (**B** and **C**), HCD (**E** and **F**), or HFD (**H** and **I**) (*N* = 6–11 mice/group; and data are shown as mean ± SEM). Statistical significance assessed by 2-tailed Student’s *t* test, *P < 0.05 denotes a significant difference from the control group on the same diet. Two-way ANOVA tests were performed for GTT and ITT, followed by Newman-Keuls multiple post hoc correction.

To test this hypothesis, we measured weight gain, glucose tolerance, and insulin sensitivity in male and female RD-fed control and TβRII^ocy–/–^ mice. Male control and TβRII^ocy–/–^ mice on RD gained minimal weight, with less than 2% increase in body weight over 16 weeks (Figure 9A), nor were genotypic differences observed in glucose tolerance between the RD-fed male control and TβRII^ocy–/–^ mice (Figure 9B). However, male TβRII^ocy–/–^ mice showed enhanced insulin sensitivity compared with the control littermates, even on the regular chow diet (Figure 9C). Female TβRII^ocy–/–^ mice on RD were heavier and had higher basal blood glucose levels than female controls (Supplemental Figure 7) but showed no genotype-dependent differences in glucose or insulin tolerance. Though the current study focuses on male mice, the sexually dimorphic metabolic phenotype of TβRII^ocy–/–^ mice motivates ongoing detailed analysis.

When fed HCD, male control mice gained weight, with a 15% increase in body weight over 16 weeks. HCD-fed TβRII^ocy–/–^ mice gained less body weight, with only a 2% increase in body weight (Figure 9D). Furthermore, TβRII^ocy–/–^ mice on the HCD showed superior blood glucose clearance tested with glucose and insulin tolerance tests (GTT and ITT) compared with control littermates (Figure 9, E and F). On the HFD, TβRII^ocy–/–^ mice again gained considerably less body weight (46%) than control counterparts (70%) (Figure 9G). HFD impaired glucose tolerance and insulin sensitivity in control mice, but HFD-fed TβRII^ocy–/–^ mice showed glucose intolerance while maintaining insulin sensitivity (Figure 9, H and I). This suggests that DMP1-Cre–mediated ablation of TβRII mitigates HFD-induced insulin resistance.

**Figure 10:**
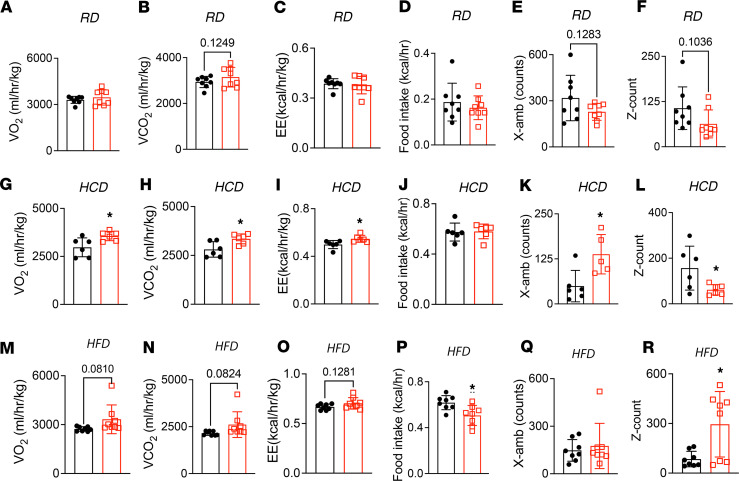
TβRII^ocy–/–^ mice exhibit metabolic protection against the effects of high-carbohydrate and high-fat diet by targeting energy expenditure, activity, and food intake. Indirect calorimetry was used to measure oxygen consumption (VO_2_), carbon dioxide production (VCO_2_), energy expenditure (EE), respiratory exchange ratio (RER), food intake, and activity (X-amb reflecting moving and exploring in the XY plane and Z-count reflecting jumping and grooming) in male control and TβRII^ocy–/–^ mice fed standard chow diet (RD, **A**–**F**), low-fat/high-carbohydrate diet (HCD, **G**–**L**), or high-fat diet (HFD, **M**–**R**) diet for 18 weeks (*N* = 6–8 mice/group; data are shown as mean ± SEM). Statistical significance, *P < 0.05, was assessed by 2-tailed Student’s *t* test.

We performed metabolic cage profiling to investigate why TβRII^ocy–/–^ mice gained less weight on HCD and HFD. On RD, no differences were observed in food intake, respiratory exchange ratio (RER), energy expenditure, or activity levels between TβRII^ocy–/–^ mice and control littermates (Figure 10, A–F, and Supplemental Figure 6, A–H). However, with HCD feeding, TβRII^ocy–/–^ mice showed higher volume O_2_ consumption, volume CO_2_ production, and energy expenditure compared with control mice, mainly at night (Figure 10, G–I, and Supplemental Figure 6, I–L). Cumulative food intake was unchanged in HCD-fed TβRII^ocy–/–^ mice, but their ambulatory movement (reflecting moving and exploring in the XY plane) was drastically higher than in control mice (Figure 10, J–L, and Supplemental Figure 6L). This suggests that the reduced weight gain in the TβRII^ocy–/–^ mice on HCD could arise from increased energy utilization and higher physical activity levels.

On HFD, TβRII^ocy–/–^ mice showed a trend toward slightly increased VO_2_ consumption, increased VCO_2_ production, and higher energy expenditure compared with HFD-fed control littermates (Figure 10, M–O, and Supplemental Figure 6, Q–U). Surprisingly, food intake was considerably reduced in HFD-fed TβRII^ocy–/–^ mice, and they continued to demonstrate increased physical activity (reflecting jumping and grooming activities), especially at night (Figure 10, P–R, and Supplemental Figure 6, S and V–X). This indicates that reduced weight gain in the TβRII^ocy–/–^ mice on HFD could arise from reduced food intake, increased energy utilization, and higher physical activity levels. Interestingly, RER values indicated that glucose and carbohydrates were the primary sources of energy for both control and TβRII^ocy–/–^ mice on RD and HCD. However, on HFD, RER values decreased, indicating a fundamental change in fuel utilization to fatty acids. Nonetheless, no noticeable differences in RER were observed between the genotypes on any diet. Together, these metabolic data demonstrate that DMP1-Cre–mediated deletion of TβRII leads to improved insulin sensitivity, which could attenuate some deleterious effects of HCD and HFD on metabolism by targeting food intake and energy expenditure.

## Discussion

Although clinical studies implicate obesity and type 2 diabetes (T2D) as drivers of human bone fragility, incomplete understanding of the causal mechanisms limits therapeutic intervention. Our work highlights the key role of osteocytic TGF-β signaling in relaying the adverse effects of high-fat and high-carbohydrate diets on bone mechanics. Particularly, we show that carbohydrate- and fat-enriched diets induce osteocyte-intrinsic TGF-β signaling to hyperactivate PLR, which maintains bone quality. Along with intensely haywire LCN and increased canalicular density, elevated TGF-β signaling induced by high fat and high carbohydrate disrupts mitochondrial function and triggers senescence in osteocytes, all of which lead to poor bone quality. Ablating osteocytic TGF-β signaling moderates responses to high-fat and high-carbohydrate diets by mitigating their effects on PLR, senescence, and functionally relevant mechanical properties such as yield stress. In addition to bone phenotype, mice deficient in osteocytic TGF-β signaling had improved metabolism in response to high-fat and high-carbohydrate diets, in part due to high energy expenditure, physical activity, and food intake. Taken together, our study supports the potential to therapeutically calibrate osteocyte function to restore impaired skeletal health and energy balance in chronic metabolic diseases, at least in part through a TGF-β–dependent mechanism.

The pathophysiological mechanisms driving skeletal fragility in metabolic conditions of obesity and T2D are complex. Several studies have used chronic administration of an HFD in rodents to model the etiology of developing obesity and T2D in humans (11–13). In such studies, choosing the correct control diet is instrumental as it can dramatically alter the findings of the study. While the choice of control diets has always been controversial, it is evident that many studies have used RD and LFDs as control diets. LFDs, although compositionally well matched to HFDs, have high amounts of carbohydrates and low fiber compared with the RD (56). Thus, a typical LFD compensating for fat calories with carbohydrates is actually an HCD, which can have substantial effects of its own when compared with RD. In fact, a few studies have reported stark phenotypic differences in mice fed RD versus LFD/HCD, including food consumption, lumbar fat mass, vascular function, and coagulation activity (56–58). Regarding bone quality, a recent study speculated that LFD/HCD may contain higher levels of advanced glycation end products and could be detrimental for bone material properties, based on the increased blood glucose levels approaching a prediabetic range in LFD mice (59). We believe that our study, for the first time, provides evidence to support the notion that LFD/HCD is detrimental to bone mechanics and causes fragility without altering bone mass. Thus, low fat content alone should not be considered a control diet. Deeper understanding of control diet contents (LFD/HCD vs. RD) is essential to delineate novel mechanisms induced by varying dietary components that contribute to bone fragility.

TGF-β, a secreted cytokine that regulates bone ECM production and remodeling, is a well-known participant in the pathophysiology of obesity and T2D (19–21). Serum levels of TGF-β are elevated in patients with obesity and T2D and in rodent models of obesity (16, 18, 19). Although bone is one of the largest repositories of TGF-β, the impact of obesity on TGF-β signaling in bone was unknown. In the current work using in vitro studies and dietary and genetic mouse models, we found osteocyte-intrinsic enhancement in TGF-β signaling by excessive fat or carbohydrate. The increased cellular TGF-β signaling could stem from increased cell surface presentation of TGF-β receptors linked to insulin, glucose, and lipids. In osteocytes, TGF-β signaling has been previously reported to cell-intrinsically stimulate regulators of PLR (38, 39). Consistent with this finding, we observed upregulation of different panels of genes implicated in PLR in response to high-carbohydrate (Mmp13, Ctsk, Atp6v1g1, and Atp6v0d2) and high-fat (Mmp14 and Atp6v0d2) diet feeding. Although this coordinated induction of PLR genes was found to be TGF-β dependent in vitro, our in vivo findings are less clear. In fact, our in vivo data hint at a partial dependency on TGF-β and that TGF-β ablation could serve to recalibrate the exacerbated PLR gene signature to the ranges detected in RD-fed control mice. Similarly, the high-carbohydrate– and high-fat–induced expansion of the LCN, which also serves as a hallmark of deregulated PLR, was found to be recalibrated to homeostatic levels through osteocytic TβRII ablation (no significant difference was found in LCN area and canalicular number between RD control mice versus HCD- and HFD-fed TβRII^ocy–/–^ mouse bones, Figure 7, A and B). Importantly, increased number of pores was apparent in the reconstructed μCT images of HCD- and HFD-fed TβRII^ocy–/–^ mice. We speculate that the diets affect the geometry of the TβRII^ocy–/–^ bones rather than the mass, and how this change in geometry contributes to the bone quality of obese TβRII^ocy–/–^ bones will be important to understand.

In this study, we aimed to examine osteocytic mechanisms by which HFD promotes bone fragility. While mitochondrial dysfunction is a recognized driver of HFD-induced cell injury and cell death in other tissues (44, 46–48), the extent to which mitochondrial dysfunction drives HFD-induced bone fragility remained untested. At the cellular level, TGF-β regulates the mitochondrial activity of endothelial cells, hepatocytes, and T cells (50, 60, 61), but its role in HFD-induced mitochondrial dysfunction in osteocytes has not been assessed to date. We find that HF and HG are sufficient to suppress osteocyte mitochondrial function. Further, we implicate the TGF-β pathway in osteocytes as a mediator of HF- and HG-mediated mitochondrial dysfunction. Therefore, blocking the TGF-β pathway mitigates some of the effects of nutrient excess on mitochondrial oxidation, as well as PLR and bone quality.

Accompanying mitochondrial dysfunction, increased osteocytic senescence was also observed in both LFD- and HFD-fed mouse bones, as gathered from the markedly elevated p16^ink4A^, p21, and p53 protein levels, which are known cell cycle arrest proteins induced by cellular stress. While an extensive characterization of cellular senescence is lacking in our study, several studies have established that metabolic distress associated with obesity and T2D promotes cellular senescence in bone (62–64). Previous studies focusing on characterizing HFD-induced obesity in postmenopausal women have shown that obesity, alone and in combination with estrogen deficiency, greatly increases the burden of senescent cells in bone (65). Similarly, the HFD-driven senescence in bone has been corroborated in males and possibly contributes to obesity-impaired fracture healing and bone fragility (64). Although one of the studies by Farr et al. did not detect increased cellular senescence in HFD-fed mouse bones (63), it is possible that the use of LFD as a control diet is responsible for this discrepancy. As evident from our studies, prolonged administration of the LFD/HCD also increased the expression of senescent markers in bone.

The TGF-β pathway is a critical determinant of systemic energy balance, and previous research in Smad 3 global knockout has demonstrated that the loss of TGF-β signaling can protect against diet-induced obesity and insulin resistance (20, 21). Similarly, the administration of a TGF-β neutralizing antibody (ID11) improves glucose tolerance, insulin sensitivity, body weight gain, and energy balance in both genetic and diet-induced obesity models (19). These metabolic improvements are attributed to increased pancreatic islet β cell function and increased browning of white adipose tissue. Our study corroborates these findings and demonstrates that the DMP1-Cre–mediated deletion of TβRII, which impairs osteocyte TGF-β signaling, can also improve metabolic outcomes by establishing energy balance under metabolic load due to excess dietary carbohydrate and fat. When fed an HCD or HFD, TβRII^ocy–/–^ mice exhibited a lean phenotype, increased energy expenditure, high physical activity levels, and reduced food intake relative to genotype controls. The increased energy expenditure in TβRII^ocy–/–^ mice might be due to changes in adipogenesis, which is regulated by sclerostin (SOST), a direct target of TGF-β signaling (66). Reduced SOST can decrease fat mass and increase insulin sensitivity (53). Although the reason for the increased physical activity in TβRII^ocy–/–^ mice remains unclear, note that our findings align with epidemiological studies revealing a correlation between low serum TGF-β levels and increased physical activity (67, 68). The differences in food intake in the TβRII^ocy–/–^ mice imply that the improved metabolic phenotype involves regulation of neuroendocrine cytokines that control appetite by signaling to the brain. One limitation of the current study is that DMP1-Cre can recombine floxed alleles in cell types other than osteocytes (69–72). In addition to thorough examination of skeletal and extraskeletal recombination of the floxed TβRII allele, future studies should identify osteokines communicating with fat, muscle, and brain that are regulated by osteocytic TGF-β signaling. These could be targets for treating obesity-induced energy imbalance.

In conclusion, this work supports several key concepts. First, just as for osteoclasts or osteoblasts, calibration of osteocyte-mediated PLR is critical for bone homeostasis. Both high-fat and high-carbohydrate diets perturb this balance in osteocytic PLR, which can substantially impair bone’s ability to resist fractures. High-fat and high-carbohydrate diets associated with poor bone mechanics and fragility also stem from suppressed osteocyte mitochondrial function and senescence. Calibrating TGF-β levels in osteocytes is important for osteocyte health since TGF-β regulates all 3 biological processes, PLR, mitochondrial function, and senescence, in osteocytes. This connection between TGF-β signaling, osteocyte health, PLR, and bone quality has been recently shown to hold true also in the context of aging (73). In addition to bone health, calibrating TGF-β levels in osteocytes could serve to reduce the metabolic burden imposed by high fat and high carbohydrates, which could further protect against bone fragility. Last, while diets rich in saturated fats are believed to be a major driving factor for obesity-associated skeletal etiologies, careful consideration must be given to the dietary contents and components, especially regarding a carbohydrate-enriched diet that can be equally deleterious for skeletal health.

## Methods

### Sex as a biological variable.

We do consider sex as an important biological variable in this study. This study mostly uses males, but some aspect of data has been collected in females that indicates the effects of osteocytic TGF-β signaling on bone fragility and metabolism during obesity could be sexually dimorphic.

### Mice.

Osteocyte-specific TβRII-deficient mice and littermate controls were generated by breeding TβRII-floxed (TβRII^fl/fl^) mice with 9.6 kb DMP1-Cre mice (38, 39). DMP1-Cre^+/–^ TβRII^fl/fl^ (TβRII^ocy–/–^ mice) and DMP1-Cre^–/–^ TβRII^fl/fl^ littermates (control) mouse lines were obtained for the subsequent diet studies. Male 12-week-old TβRII^ocy–/–^ and control mice were fed a regular chow diet (Pico Lab Mouse Diet 20, 22% kcal fat, *N* = 8–10, RD group), a high-fat diet (Research Diets D12492, 60% kcal fat, *N* = 8–12, HFD group), or the recommended control low-fat but carbohydrate-enriched diet (D12450J Research Diets, 10% kcal fat, *N* = 8–12, HCD group) for 18 weeks. Male mice were used in this study because female mice showed no genotype-dependent change in bone quality or osteocyte function in basal conditions (39). Additional details for the mouse genotyping procedure are described in Supplemental Methods.

### μCT.

For skeletal phenotyping, femurs harvested from 30-week-old male TβRII^ocy–/–^ and control mice fed HFD, HCD, or RD were harvested and cleaned of the surrounding soft tissue, fixed in 10% neutral buffered formalin for 48 hours, and stored in 70% ethanol for μCT. μCT procedure details are provided in Supplemental Methods (38, 39, 73).

### Flexural strength tests.

Whole-bone mechanical properties were determined by 3-point bending of intact, hydrated right femurs isolated from 30-week-old male TβRII^ocy–/–^ and control mice (*N* = 7–11 mice) from each diet group. Details of the procedure are described in Supplemental Methods (38, 39, 73).

### SRμCT.

SRμCT studies were used to assess the degree of bone mineralization and morphological parameters of osteocyte lacunae and vasculature. The femoral mid-diaphysis of 30-week-old male mice of both genotypes that were fed RD, HCD, and HFD were scanned with a 20 keV x-ray energy, a 300 ms exposure time, and a 4× magnifying lens for a spatial resolution of 1.3 μm (*N* = 4 bones/group) (74, 75). Additional details of the SRμCT procedure are described in Supplemental Methods.

### Cell culture.

Undifferentiated OCY454 cells were seeded at 1 × 10^5^ to 1.2 × 10^5^ cells/well density in 6-well plates and allowed to become confluent in media consisting of αMEM (Gibco; 12571-063), 1% Antibiotic-Antimycotic (Gibco; 15240062), and 10% heat-inactivated fetal bovine serum (Gibco; 10437-028) at 33°C before being differentiated at 37°C for 3 days (76). Differentiated OCY454 cells were serum-starved for 1 hour and then treated for 72 hours with SB431542 (10 μM, Selleckchem), glucose (25 mM, MilliporeSigma) for HG treatment, or the BSA-conjugated fatty acids palmitate (100 μM, Cayman Chemicals), oleate (200 μM, Cayman Chemicals), and linoleate (200 μM, MilliporeSigma) for HF treatment. Details of cell culture are described in Supplemental Methods.

### qRT-PCR.

Total RNA was extracted and purified from cleaned humeri devoid of marrow and epiphyses using the miRNeasy Mini Kit (QIAGEN) and subjected to cDNA synthesis and qRT-PCR (38, 39). mRNA levels were deduced from absolute quantification of gene expression, and 18s ribosomal RNA was used as an internal reference gene. Sequences of the primers have been provided in Supplemental Table 1. Details of qRT-PCR procedures are described in Supplemental Methods.

### Immunoblotting analysis.

Whole-cell lysates of OCY454 cells were collected in RIPA lysis buffer (50 mM Tris pH 7.4, 1% NP-40, 0.25% sodium deoxycholate, 150 mM NaCl, 1 mM EDTA, phosphatase inhibitor, and protease inhibitor). Lysates were sonicated and centrifuged at 10,000*g*, 10 minutes, at 4°C. Then 25 μg of protein was loaded onto 10% SDS-PAGE. Protein was then transferred onto the nitrocellulose blot, blocked, and incubated in the primary antibodies overnight for phospho–Smad 3 (1:2,000, ab52903), p16^ink4a^ (1:1,000, ab211542), p53 (1:1,000, ab227655), and β-actin (1:2,500, ab8226) and IRDye fluorophore-conjugated secondary antibodies (1:15,000, LI-COR Biosciences). Bands’ intensity corresponding to protein of interest were visualized, quantified, and normalized to β-actin protein. Fold-change in protein relative to untreated control is presented as mean ± SD. Our sample size was 5 biological replicates/group, achieved by compiling 3 independent experiments.

### Seahorse extracellular flux assay.

Undifferentiated OCY454 cells were seeded into specialized XFe24 V7 microplates at 20,000 cells/well density, serum-starved, and then treated with TGF-β (5 ng/mL, PeproTech), SB431542 (10 μM, Selleckchem), glucose (25 mM, MilliporeSigma), or the BSA-conjugated fatty acids palmitate (100 μM, Cayman Chemicals), oleate (200 μM, Cayman Chemicals), and linoleate (200 μM L9530-5ML, MilliporeSigma) for 24 hours. Mitochondrial and glycolytic stress tests were performed to determine the OCR and ECAR, which were then normalized to the cell lysate protein in each well. Data were obtained from 3 independent biological experiments, with 5–6 technical replicates in each experiment. Details are described in Supplemental Methods.

### Cellular ROS analysis.

Intracellular ROS levels were measured using the DCFDA/H2DCFDA kit (Abcam, ab113851) according to the manufacturer’s instructions. Cells were stained with 20 μM DCFDA in 1× buffer for 30 minutes at 37°C, washed in 1× buffer, and analyzed by flow cytometry on the LSR Fortessa instrument (BD Biosciences) using the FACSDiva software (BD) and FlowJo software to generate plots. In each experiment, 3 technical replicates were used, and reproducible data from 3 independent experiments are reported.

### Mitochondrial membrane potential analysis.

Mitochondrial membrane potential was evaluated with the potentiometric dye JC-1 (5,5′,6,6′-tetrachloro-1,1′,3,3′-tetraethyl benzimidazole-carbocyanine iodide). Cells were seeded on collagen-coated glass-bottom MatTek dishes (BICO), treated for 24 hours, and then incubated with JC-1 (5 mg/mL) for 30 minutes prior to live cell imaging (5% CO_2_, 37 °C) conducted on a ZEISS Airyscan microscope with a 63×/1.4 oil objective. Data were reproducible across 3 independent biological experiments conducted by 2 individuals, with 3–4 images collected for each treatment in the individual experiment. Additional details are in Supplemental Methods.

### Histology, immunofluorescence, and immunohistochemistry.

Femurs were decalcified in 10% EDTA for 28 days and processed for paraffin embedding. Seven micrometer–thick axial sections of femoral cortical bone were used for immunohistochemistry and Ploton silver stain. Primary antibodies were anti-p16^ink4a^ antibody (1:50, ab211542, Abcam), anti-p53 antibody (1:75, ab227655, Abcam), rabbit polyclonal anti-p21^cip1/waf1^ (1:50, 2947, Cell Signaling Technology), and anti-γH2A.X (1:50, ab11174, Abcam). Subsequently, sections were incubated with a secondary antibody for 2 hours, and the signal was amplified with Alexa Fluor 594 tyramide (Tyramide SuperBoost Kit, Invitrogen). The presence of autofluorescence or nonspecific reactivity was ruled out by taking as reference a sample incubated with rabbit IgG primary antibody. Quantification of osteocyte LCN and immunofluorescence were performed as previously described (38, 39), with 4 high-power fields/mouse bone and *N* = 5 mice/group. Details of these procedures are described in Supplemental Methods.

### GTT and ITT.

GTT and ITT were administered after mice were fed an RD, HCD, or HFD for 18 weeks. Mice were fasted overnight for GTT and 4 hours for ITT, following which glucose (1.5 mg/kg of body weight) or insulin (0.75 U/kg of body weight, Humulin, Lilly USA) was administered intraperitoneally, and blood glucose levels were measured at 0, 15, 30, 60, and 120 minutes after injection with an Accu-Chek Aviva meter (Roche Diabetes Care, Inc.).

### Indirect calorimetry.

Energy expenditure was evaluated by indirect calorimetry and ambulation (activity) using the automated Comprehensive Lab Animal Monitoring System (Columbus Instruments) at the University of California, San Francisco. Acclimation, measurement, and housing protocols were based on previously published studies (77), and additional details are provided in Supplemental Methods.

### ELISA.

Active TGF-β1 levels in the serum were measured using the mouse TGF-β1 DuoSet ELISA (DY1679-05) (R&D Systems, Bio-Techne), following the manufacturer’s protocol.

### Statistics.

The sample size was determined based on a power calculation that provides an 80% chance of detecting a significant difference (*P* < 0.05). Technical replicates and biological replicates (*N*) used for all experiments are described in the figure legends. Where technical replicates are used, data are expressed as mean ± SEM. Otherwise, data are reported as mean ± SD. Prism 5.0 (GraphPad Software, Inc.) was used for statistical analysis. Student’s 2-tailed *t* tests were used when determining the statistical differences between the 2 groups; while comparing multiple groups, we used 1-way ANOVA followed by the Newman-Keuls test for multiple comparisons. To analyze the effect of 2 variables and the interaction of those variables, we used 2-way ANOVA followed by the Newman-Keuls test for multiple comparisons.

### Study approval.

Mice were housed in groups in a pathogen-free facility at 22°C with a 12-hour light/12-hour dark cycle and fed their respective diets (irradiated) and water ad libitum. All studies were conducted with the approval of the Institutional Animal Care and Use Committee of the University of California, San Francisco.

### Data availability.

All data associated with this study are present in the paper or the supplemental information, and raw data are included in the Supporting Data Values file.

## Author contributions

NSD and TA conceived the studies, procured funding, planned the experiments, and provided project leadership and supervision. NSD, ABT, SK, YO, CAS, JY, CSY, VK, CAL, MC, JLS, EM, and CA collected and analyzed data. NSD and TA interpreted data and prepared the manuscript, which was edited and approved by all authors.

## Supplementary Material

Supplemental data

Unedited blot and gel images

Supporting data values

## Figures and Tables

**Table 1:**
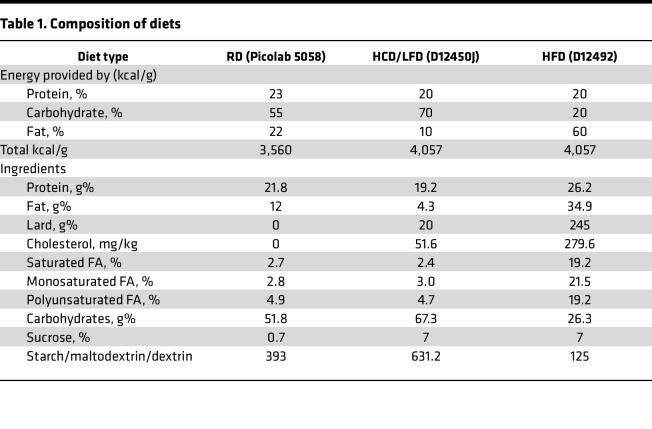
Composition of diets

**Table 2:**
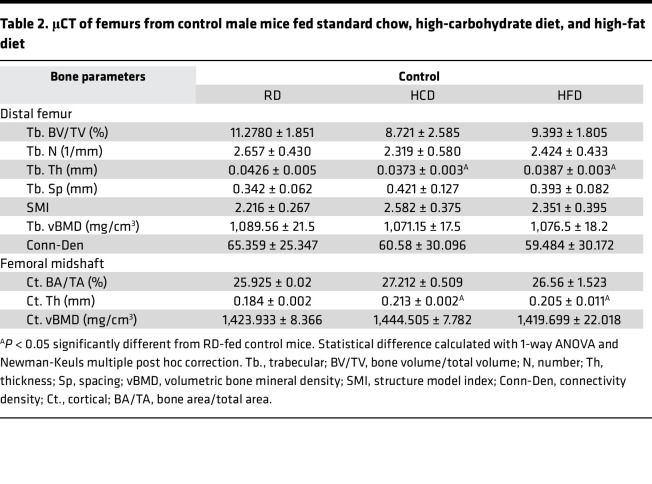
μCT of femurs from control male mice fed standard chow, high-carbohydrate diet, and high-fat diet

**Table 3:**
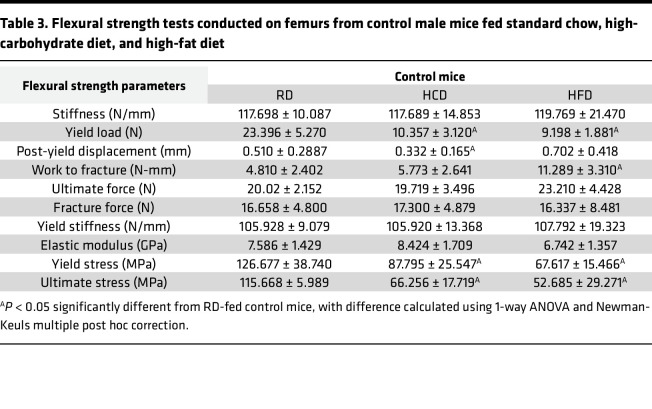
Flexural strength tests conducted on femurs from control male mice fed standard chow, high-carbohydrate diet, and high-fat diet

**Table 4:**
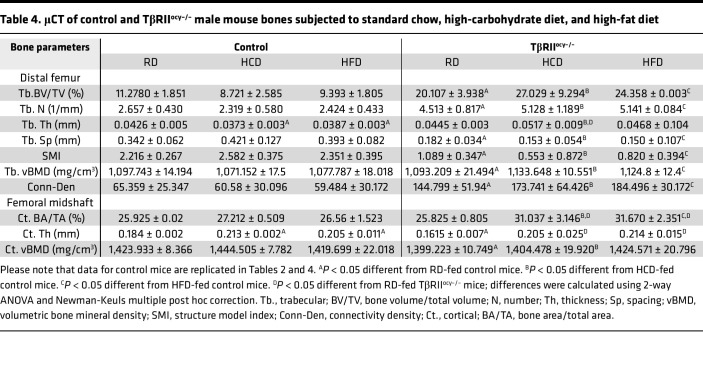
μCT of control and TβRII^ocy–/–^ male mouse bones subjected to standard chow, high-carbohydrate diet, and high-fat diet

**Table 5:**
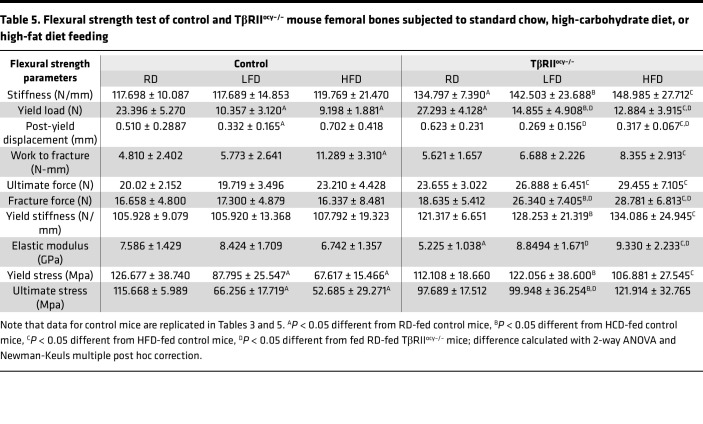
Flexural strength test of control and TβRII^ocy–/–^ mouse femoral bones subjected to standard chow, high-carbohydrate diet, or high-fat diet feeding

## Version 1 08/22/2024

Electronic publication
