# Supplementary material for: High-fat and high-carbohydrate diets increase bone fragility through TGF-β–dependent control of osteocyte function
Source: JCI Insight. 2024 Jul 9;9(16):e175103. doi: 10.1172/jci.insight.175103 (PMC11343608; doi:10.1172/jci.insight.175103)
Supplement: Supplemental data [file jciinsight-9-175103-s203.pdf]

Supplemental Material.

Methods:

Mice. Osteocyte-specific TβRII-deficient mice and littermate controls were generated by breeding TβRII floxed (TβRII $^{fl/fl}$) mice with 9.6kb Dmp1-Cre mice (33, 34). DMP1-Cre$^{+/-}$; TβRII$^{fl/fl}$ (named TβRII$^{ocy-/-}$ mice) and DMP1-Cre$^{-/-}$; TβRII fl/fl littermate controls (named Control) mouse lines were obtained for subsequent experiments. Genotyping was conducted with 50 ng of genomic DNA from tail biopsies. The presence of a Dmp1-Cre coding sequence was confirmed using forward 5'-TTG CCT TTC TCT CCA CAG GT-3' and reverse 5'-CAT GTC CAT CAG GTT CTT GC-3' primers. The presence of a floxed allele was confirmed using forward 5'-TAT GGA CTG GCT GCT TTT GTA TTC-3' and reverse 5'-TGG GGA TAG AGG TAG AAA GAC ATA-3' primers. All transgenic mouse lines were backcrossed into a congenic C57BL/6 background for eight generations.

For diet studies, 12-week-old male TβRII$^{ocy-/-}$ and control mice were fed either a regular chow diet (Pico Lab Mouse Diet 20, 22% kcal fat, 55% kcal carbohydrates, 23% kcal protein, N=8-10, RD group), high-fat diet (Research Diets D12492 60% kcal fat, 20% kcal carbohydrates 20% kcal protein, N=8-12, HFD group), or the recommended 'control' low-fat but carbohydrate enriched diet (D12450J Research Diets 10% kcal fat, 70% kcal carbohydrate, 20% kcal protein, N=8-12, HCD group) for 18 weeks. Male mice were used in this study because female mice showed no genotype-dependent change in bone quality or osteocyte function in basal conditions (34). Mice were housed with their groups (4-5 per cage) in a pathogen-free facility at 22^{0}C with a 12-hour light/dark cycle and fed their respective diets ad libitum. All diets were irradiated, and mice were allowed to drink water ad libitum. All studies were conducted with the approval of the Institutional Animal Care and Use Committee of the University of California San Francisco.

Micro CT analysis. For skeletal phenotyping, left femurs from 30-week-old male TβRII$^{ocy-/-}$ and control mice fed either a high-fat diet (HFD), low-fat/high-carbohydrate diet (HCD), or regular diet of standard chow (RD) were harvested and cleaned of the surrounding soft tissue. Cleaned bones were subsequently fixed in 10% neutral buffered formalin and stored in 70% ethanol. A 2 mm region of the femoral metaphysis and a 1 mm region of the femoral mid-diaphysis were scanned using a Scanco µCT50 specimen scanner to assess trabecular and cortical parameters, respectively. Scanning was performed with an X-ray potential of 55 kVp and a current of 109 µA at a resolution of 10 µm. After scanning, scan projections were reconstructed to generate cross-sectional images using a cone-beam reconstruction algorithm. Density equivalent values are measured by calibrating the scanner to a hydroxyapatite phantom provided by the manufacturer. The trabecular bone compartment (300 µm proximal to epiphyseal plate) was segregated using manual delineation of the endosteal surface and morphometry was characterized by measuring the bone volume fraction (BV/TV), trabecular thickness (Tb. Th.), trabecular number (Tb. N.), and trabecular spacing (Tb. Sp.). Cortical morphometry was quantified within a 500 µm mid-diaphyseal span (50 serial sections) centered at the mid-point between proximal and distal epiphyses. Cross-sectional measurements included cortical bone area (Ct. Ar.), thickness (Ct. Th.), and cortical bone mineral density (Ct. tBMD) (33, 34, 40).

Synchrotron Radiation Micro-Tomography (SRµCT). SRµCT studies were used to assess the degree of bone mineralization and morphological parameters of osteocyte lacunae and vasculature. The femoral mid-diaphysis of 30-week-old male mice of both genotypes that were fed RD, HCD, and HFD diets were scanned with a 20 keV x-ray energy, a 300 ms exposure time, and a 4x magnifying lens for a spatial resolution of 1.3 µm (n = 4 bones/group). Imaging was performed at the Advanced Light Source on Beamline 8.3.2 at the Lawrence Berkeley National Laboratory by obtaining two-dimensional radiographs as the specimens were rotated from 0° to 180°. The radiographs were reconstructed in 3D Fourier-filtered back projection using the

TomoPy reconstruction package in Python (41). The attenuation coefficient or gray values for each voxel is divided by the mass attenuation coefficient of bone (4.001 cm2/g at 20 keV) to directly calculate bone mineral density. Dragonfly (Object Research Systems (ORS) Inc, Montreal, Canada, version 2022.2) image analysis software was used to filter, segment, and quantify the canals and lacunar features (42). After adjusting brightness and contrast uniformly and converting the images to 8-bit, a grayscale threshold of 160 was used to segment porosities within each sample. The intersection of a bone mask and the porosities was taken to separate canals from the background and a particle filter was used to exclude porosities less than ~4400 μm^3 in size to obtain the vascular canal segmentations.

Flexural strength tests. Whole-bone mechanical properties were determined by three-point bending of intact, hydrated right femurs isolated from 30-week-old male $T\beta RII^{ocy-/-}$ and control mice (N=7-11 mice) from each diet group. Immediately prior to testing, the femur was brought to room temperature and placed in HBSS. The bone was centered over an 8-mm lower span (40% average femur length) with the anterior side facing down so that the anterior diaphysis was loaded in tension. Three-point bending was performed to failure using an actuator speed of 0.01 mm/sec (EnduraTec ELF 3220, Bose Corp., Minnetonka, MN). Force (500-g capacity load cell, Sensotec Model 31/6775-06, Honeywell Sensotec, Columbus, OH) and displacement were recorded at 100 Hz. After failure, the femur was immediately wrapped in HBSS-soaked gauze and returned to −20°C. Yield load, maximum load, stiffness, post-yield displacement (PYD), and work-to-fracture were calculated from load-displacement curves with a custom written MATLAB® script (R2017, The MathWorks, Inc., Natick, MA). Yield was calculated as the point where a line with a 10% decrease in stiffness intersected the force-displacement curve. PYD was calculated as the difference between the displacement at yield and the displacement at failure. The fracture surface of the femurs was imaged using scanning electron microscopy (ZEISS Sigma 500 VP FE-SEM)

under an excitation voltage of 15kV and various pressure settings. The moment of inertia was determined by measuring the endosteal and periosteal cross-sectional diameters using ImageJ, assuming an elliptical cross-section, which was then used to calculate the stress-strain curve. The apparent material properties, including yield stress, maximum stress, and Young's elastic modulus, were derived from the estimated stress-strain curves with MATLAB® (33, 34, 40).

Cell culture. Undifferentiated OCY454 osteocyte-like cells, gifted by P. Divieti Pajevic, were cultured on collagen-coated (Corning; 354236) plates with αMEM (Gibco; 12571-063), 1% Anti-Anti (Gibco; 15240062), and 10% heat-inactivated fetal bovine serum (Gibco; 10437-028) at 33°C (43). OCY454 cells were seeded at 1-1.2x10^5 cells/well density in 6-well plates and allowed to become confluent before being differentiated at 37^0C for three days. Differentiated OCY454 cells were serum starved for 1 hr and then treated for 72 hr with SB431542 (10 μM, Selleckchem), glucose (25 mM, Sigma) for high glucose treatment (HG), or the BSA-conjugated fatty acids palmitate (100 μM, Caymen Chemicals), oleate (200 μM, Caymen Chemicals), and linoleate (200 μM L9530-5ML, Sigma) for high-fat treatment (HF). All treatments were prepared in 0.5% FBS-αMEM media. At the end of the treatment, samples were processed for either qRT-PCR or immunoblotting outcomes.

Seahorse extracellular flux assay. Undifferentiated OCY454 cells were seeded into specialized XFe24 V7 microplates (Seahorse Bioscience) at 20,000 cells/well density and cultured overnight before treatments. Cells were serum starved for 1 hr and then treated with TGFβ (5ng/ml, Peprotech), SB431542 (10 μM, Selleckchem), glucose (25 mM, Sigma), or the BSA-conjugated fatty acids palmitate (100 μM, Caymen Chemicals), oleate (200 μM, Caymen Chemicals), and linoleate (200 μM L9530-5ML, Sigma) for 24 hr. Treatments were prepared in 0.5% FBS- αMEM media. The treated cells were exposed to different experimental conditions, and the oxygen consumption rate (OCR) and extracellular acidification rate (ECAR) were measured using a

Seahorse Bioscience Extracellular Flux Analyzer (Agilent). To measure OCR, cells were treated sequentially with oligomycin (Millipore 495455-10MG; 2 μM), FCCP (Sigma C2920-10MG; 2 μM), Rotenone (Fluka 45656-250MG 1 μM), and Antimycin A (Sigma A8674-25MG; 1 uM), while ECAR measurement was conducted in the Seahorse XFe24 Analyzer by sequentially treating cells with glucose (catalog #; 5.5 mM), oligomycin (Millipore 495455-10MG; 2 μM), and 2-deoxyglucose (Sigma D8375-5G; 50 mM). The cells were then lysed in RIPA buffer and subjected to a protein assay. The OCR and ECAR values were normalized to the protein values in each well in Wave Desktop software, with 5-6 technical replicates. Data was concluded from 3 independent biological experiments, and representative data from one of the experiments has been shown.

Cellular ROS analysis. Intracellular ROS levels were measured using the DCFDA/H2DCFDA kit (Abcam, Cat#ab113851) according to the manufacturer's instructions. Cells were stained with 20μM DCFDA in 1X buffer for 30 min at 37°C, washed in 1X buffer, and analyzed by flow cytometry on the LSR FORTESSA instrument (BD Biosciences, San Jose, CA) using the FACS Diva software (BD). The final analysis, including the generation of plots and histograms, was performed using FlowJo software v.10 (BD). In each experiment, 3 technical replicates were used, and reproducible data from 3 independent experiments is reported.

Mitochondrial membrane potential analysis. Mitochondrial membrane potential (DYm) was evaluated with the potentiometric dye JC-1 (5,5′,6,6′-tetrachloro-1,1′,3,3′- tetraethyl benzimidazole-carbocyanine iodide; Carlo Erba). Cells were seeded on collagen-coated glass bottom (35 mm) MatTek dishes at 40,000 cells per dish, allowed to attach overnight, and then treated for 24 hours. The media was then replaced with fresh media containing JC-1 (5mg/ml), and cells were incubated in the dark for an additional 30 minutes at 37°C. After the JC1 media was removed, cells were washed and incubated in reduced serum media (α-MEM supplemented

with 0.5% FBS). Confocal z-stacks were acquired in a chamber equipped for live cell imaging (5% CO_2, 37 °C) using a Zeiss Airyscan microscope with the 63x/1.4 oil objective. Analysis used ImageJ with sum projections of red and green channels. Red-to-green ratios (R/G) were calculated by dividing the red and green intensity. These values were then normalized to the control condition, and fold change in the mean R/G fluorescence was shown. For each treatment, 3-4 images were collected in each experiment, and representative data from one of the experiments was shown. Our data was reproducible across 3 independent biological experiments conducted by two individuals.

Immunoblotting Analysis. Whole-cell lysates of OCY454 cells were collected in RIPA lysis buffer containing 50 mM Tris pH 7.4, 1% NP-40, 0.25% sodium deoxycholate, 150 mM NaCl, 1 mM EDTA, supplemented with phosphatase inhibitor (A32957, Pierce), protease inhibitor (cOmplete Mini, Roche), and 1 mM PMSF. Lysates were sonicated on ice using a cup horn sonicator (five 15-second pulses, 45 seconds between pulses) and cleared by centrifugation at 10,000×g for 10 minutes at 4°C. Protein concentration was determined using the Pierce BCA Protein Assay Kit (ThermoScientific; 23225). 25 μg of total lysate from each sample was loaded onto 10% SDS-polyacrylamide gels, separated, transferred to a nitrocellulose membrane, and blocked in 5% milk in Tris-buffered saline (TBS) with 0.1% Tween 20. Subsequently, blots were probed with anti-phospho-Smad3 (1:2000, rabbit, ab52903, Abcam), anti-$p16^{ink}$ antibody (1:1000, rabbit, ab211542, Abcam), anti-p53 antibody (1:1000, rabbit, ab227655, Abcam), and anti-β-actin (1:2500, mouse, ab8226, Abcam) antibodies, all of which were prepared in 5% of BSA, TBS with 0.1% of Tween 20. Anti-mouse and anti-rabbit secondary antibodies conjugated to 680 or 800 IRDye fluorophores (1:15000, LI-COR Biosciences) were used. After probing with secondary antibodies, band intensities were visualized using an Odyssey infrared imaging system (LI-COR Biosciences) and quantified using Image Studio Lite (v5.2, LI-COR Biosciences). Please note that

blots were stripped and re-probed for different antibodies. The efficiency of the stripping protocol was verified by the absence of any secondary antibody binding to the stripped blot. Fold changes were calculated relative to unstimulated controls after normalizing the signal from proteins of interest to β-actin. Fold change data is presented as the mean ± SD, and blots are representative of quantitative data collected from 3 biological replicates/groups/experiments, and data gathered from compiling 3 independent experiments has been shown. Ultimately our sample size from compiling 3 independent experiments was 5 biological replicates/ group.

Histology, Immunofluorescence, and Immunohistochemistry. Femurs from each group were decalcified in 10% di- and tetra-sodium EDTA for 28 days and then processed for paraffin embedding and sectioning. Briefly, 7 μm thick axial sections of cortical bone were used for immunohistochemistry and Ploton silver stain. Antigen retrieval was performed by incubating slides in Unitrieve (Innovex, 65^0 C-30 min), followed by 3% hydrogen peroxide (RT-10 min), blocking with 10% goat serum (RT-1hr), and background buster (Innovex, RT-1 hr). This was followed by overnight incubation with primary antibodies, namely, anti-$p16^{ink}$ antibody (1:50, rabbit, ab211542, Abcam); anti-p53 antibody (1:75, rabbit, ab227655, Abcam), and rabbit polyclonal anti-$p21^{cip1/waf1}$ (1:50, rabbit, 2947, Cell Signaling), and anti-gamma H2A.X (1:50, rabbit, ab11174, Abcam). Subsequently, sections were incubated with a secondary antibody for two hours and followed by tyramide signal amplification with Alexa Flour 594 tyramide (Tyramide SuperBoost Kit with Alexa Fluor Tyramides, Invitrogen) according to its manufacturer's protocols. DAPI was used as a nuclear stain. Sections were viewed and imaged with a Lecia DMi8 (Leica Microsystems, Wetzlar, Germany) inverted confocal microscope operating LAS X software, followed by quantification using Image J software (NIH, Bethesda, MD) in more than 4 fields per slide. The presence of autofluorescence or nonspecific reactivity was ruled out by taking as reference a sample incubated with rabbit IgG primary antibody. Sections were evaluated for the

colocalization of p16ink4A, p21cip, and p53 with DAPI was performed by employing the Coloc 2 plugins of Fiji software. The difference in dually stained cells within a bone sample from control or TβRII$^{ocy-/-}$ mice on different diets was expressed in fold change. Data was collected from 4 regions/section from 2 sections/mouse, and N=3 mice/ diet group.

To visualize and quantify the osteocyte lacunocanalicular network, Ploton silver staining was performed as previously described (33, 34). Images were acquired using a Nikon Eclipse E800 bright-field microscope (Rasband, W.S., ImageJ, U.S. National Institutes of Health, Bethesda http://imagej.nih.gov/ij/). The quantitative average represents data from 4 high-powered fields/ mouse bone and N=5 mice/group. The lacuno-canalicular network area (LCN area) was normalized to the bone area and expressed in fold change. The number of canaliculi was determined using the previously established methodology (33, 34).

Indirect calorimetry. Energy expenditure by indirect calorimetry and ambulation (activity) were evaluated using the automated Comprehensive Lab Animal Monitoring System (CLAMS, CLAMS; Columbus Instruments, Columbus, OH) at the University of California, San Francisco. The Oxymax CLAMS system software (see manufacturer's technical document, Oxymax for Windows) uses O_2 consumption (VO_2) and CO_2 production (VCO_2) to calculate the respiratory exchange ratio (RER: VCO_2/VO_2) and to calculate energy expenditure (heat production) using an equation based on RER-associated kilocalories per liter of consumed O_2: "calorific values" from Lusk. Acclimation, measurement, and housing protocols were based on previously published studies examining the effects of temperature on gas exchange in HFD-fed mice (44). Briefly, to minimize variance due to the transition of animals to the calorimetry environment and caging, mice were placed in acclimation cages (calorimeter chambers not connected to the system) and housed in the calorimeter room at 22°C for 24 hr to be further acclimated before the start of the calorimetry measurements taken. The input weight for the CLAMS energy expenditure calculation was the

weight of each mouse at the start of each temperature period. The final 72 hr of measurement at each temperature were used to calculate and report energy expenditure and energy intake.

Supplemental Tables

***Supplemental Table 1*: Primers used for SYBR qRT-PCR analysis of gene expression.**

Gene	Sequence
18s RNA (sense)<br>18s RNA (antisense)	5'-CGAACGTCTGCCCTATCAAC-3'<br>5'-GGCCTCGAAAGAGTCCTGTA-3'
Mmp13 (sense)<br>Mmp13 (antisense)	5'-CGGGAATCCTGAAGAAGTCTACA-3'<br>5'-CTAAGCCAAAGAAAGATTGCATTTC-3'
Mmp14 (sense)<br>Mmp14 (antisense)	5'-AGGAGACGGAGGTGATCATCATTG-3'<br>5'-GTCCCATGGCGTCTGAAGA-3'
Mmp2 (sense)<br>Mmp2 (antisense)	5'-AACGGTCGGGAATACAGCAG-3'<br>5'-GTAAACAAGGCTTCATGGGG-3'
Ctsk (sense)<br>Ctsk (antisense)	5'- GAGGGCCAACTCAAGAAGAA-3'<br>5'- GCCGTGGCGTTATACATACA-3'
Acp5 (sense)<br>Acp5 (antisense)	5'-CGTCTCTGCACAGATTGCAT-3'<br>5'-AAGCGCAAACGGTAGTAAGG-3'
Serpine1 (sense)<br>Serpine1 (antisense)	5'-CAGATGACCACAGCGGGGAA-3'<br>5'-GGCATGAGCTGTGCCCTTCT-3'
Atp6v0d2 (sense)<br>Atp6v0d2(antisense)	5'-TCTTGAGTTTGAGGCCGACAG-3'<br>5'-GCAACCCCTCTGGATAGAGC-3'
Atp6v1g1 (sense)<br>Atp6v1g1 (antisense)	5'-CCGTTCTCTCAGCCCAAAGT-3'<br>5'-CTCCGGTTCTTTCGCTTGC-3'
β-actin (sense)<br>β-actin (antisense)	5'-CTCTGGCTCCTAGCACCATGAAGA-3'<br>5'-GTAAAACGCAGCTCAGTAACAGTCCG-3'

***Supplemental Table 2*: TaqMan primers used for qRT-PCR analysis of gene expression.**

Gene	TaqMan probe
Rn18s	Mm03928990_g1
Tgfbr2	Mm03024091_m1
Ckdn2a	Mm00494449_m1
Cdkn1a	Mm04207341_m1
Trp53 (Tp53)	Mm01731287_m1
Serpine1	Mm00435858_m1

***Supplemental Table 3*: 2-way ANOVA comparing individual and combined effects of TGFβ ligand, fatty acids, and TGFβ pathway inhibitor (SB431542) on osteocyte energetics.**

	**p values**					
	**TGFβ factor**	**HF factor**	**TGFβ * HF Interaction**	**SB431542 factor**	**HF factor**	**SB431542* HF Interaction**
**OCR parameters**						
Non-Mito O2	0.0411	0.0604	0.5088	0.0063	0.0184	0.0233
Basal Respiration	0.0008	0.9391	0.0365	0.2297	0.0003	0.0852
Maximal Respiration	<0.0001	<0.0001	0.1401	0.0165	0.0003	<0.0001
ATP Production	<0.0001	<0.0001	0.9044	0.0006	0.0235	0.0006
Proton Leak	0.0239	0.0243	0.1877	0.9851	0.0537	0.1514
Spare Resp Capacity	<0.0001	<0.0001	0.5975	0.5006	0.1973	0.4876
**ECAR parameters**						
Non-Gly Acidification	0.2559	0.0411	0.5373	0.0013	<0.0001	0.0387
Glycolytic Reserve	0.0001	<0.0001	0.0197	0.0261	0.0003	0.0261
Glycolysis	0.7836	<0.0001	0.8263	0.1788	0.0001	0.0309
Glycolytic capacity	0.0013	<0.0001	0.0232	0.0131	0.0003	0.0006
**ROS production**						
Cellular ROS	<0.0001	<0.0001	0.0497			

***Supplemental Table 4*: 2-way ANOVA comparing individual and combined effects of TGFβ ligand, high glucose, and TGFβ pathway inhibitor (SB431542) on osteocyte energetics.**

	**p values**					
	**TGFβ factor**	**HG factor**	**TGFβ * HG Interaction**	**SB431542 factor**	**HG factor**	**SB431542* HG Interaction**
**OCR parameters**						
Non-mito O2	0.1921	0.0511	0.4922	0.0063	0.0184	0.0233
Basal Respiration	0.0303	0.0064	0.0032	0.2297	0.0003	0.0852
Maximal Respiration	0.0119	0.0037	0.0039	0.0165	0.0003	<0.0001
ATP Production	0.0293	0.0034	0.0032	0.0006	0.0235	0.0006
Proton Leak	0.1459	0.0916	0.1032	0.9851	0.0537	0.1514
Spare Resp Capacity	0.1389	0.2172	0.0625	0.5006	0.1973	0.4876
**ECAR parameters**						
Non-Glyc Acidification	0.3543	0.1886	0.1357	0.4654	<0.0001	0.0179
Glycolytic Reserve	0.003	0.0705	0.678	0.9112	0.0034	0.0116
Glycolysis	0.0352	0.0009	0.0378	0.0902	<0.0001	0.0703
Glycolytic capacity	0.0025	0.0014	0.4815	0.0065	0.0481	0.0016
**ROS production**						
Cellular ROS	<0.0001	<0.0001	0.9227			

***Supplemental Table 5*: 2-way ANOVA comparing individual and combined effects of TGFβ pathway inhibitor (SB431542), high fatty acids, and high glucose on osteocyte genes.**

p values						
**Osteocyte gene expression**	**SB431542 factor**	**HF factor**	**SB431542* HF Interaction**	**SB431542 factor**	**HG factor**	**SB431542* HG Interaction**
Serpine1	0.0063	0.0072	0.0078	0.0081	0.0184	0.0102
Mmp13	0.0399	0.0330	0.0272	0.1234	0.3465	0.0332
Mmp14	0.0076	0.0013	0.0807	<0.0001	0.0005	0.0002
Ctsk	0.0008	0.0030	0.0006	0.1080	0.2402	0.1028
Atp6v0d2	<0.0001	<0.0001	0.0003	0.0003	0.0028	0.0037
Atp6v1g1	<0.0001	<0.0001	<0.0001	0.0004	0.0054	0.0057
Cdkn2a	0.2574	0.0853	0.0054	0.8966	0.0837	0.0016
Tp53	0.0007	0.0022	0.0019	0.0144	0.1412	0.0409

***Supplemental Table 6*: 2-way ANOVA comparing individual and combined effects of ablated TGFβ signaling, HCD, and HFD diet on osteocyte gene expression, senescence markers, and lacunar-canalicular features, within bones of control and TβRII$^{ocy-/-}$ mice.**

p values						
	**TGFβ factor**	**HCD factor**	**TGFβ * HCD Interaction**	**TGFβ factor**	**HFD factor**	**TGFβ * HFD Interaction**
**Osteocyte gene expression**						
TβRII	<0.0001	<0.0001	0.0002	0.0018	0.007	0.1193
Serpine1	0.0008	0.9391	0.0365	0.006	<0.0001	0.0032
Mmp2	<0.0001	0.4322	0.7836	0.0001	0.7528	0.4464
Mmp13	0.0053	0.0002	0.6704	0.0009	0.0176	0.5621
Mmp14	0.0007	0.0385	0.6039	0.0007	0.001	0.5833
Ctsk	0.0002	0.0035	**0.0026**	<0.0001	0.0006	**0.0014**
Atp6v0d2	0.0082	<0.0001	0.4032	0.0004	<0.0001	0.8126
Atp6v1g1	<0.0001	<0.0001	**0.007**	<0.0001	0.0004	**0.005**
**Senescence markers with Immunofluorescence**						
p16ink4a IF	0.0855	<0.0001	0.0748	0.0007	<0.0001	0.0008
p21cip/waf IF	0.3556	0.0001	0.9791	0.0015	<0.0001	0.0117
p53 IF	0.1904	0.0054	0.0815	0.5191	0.0599	0.2808
**Lacunar-canalicular features**						
LCN Area	<0.0001	<0.0001	**0.012**	<0.0001	<0.0001	**<0.0001**
No of Canaliculi	<0.0001	0.0005	**0.0033**	<0.0001	<0.0001	**0.0033**
Lacunar Density	0.2233	<0.0001	0.9371	0.4471	0.0001	0.7696
Peak Lacunar Volume	0.2247	0.0073	**0.002**	0.3712	0.066	0.1568
Ca Dm	0.0002	0.4047	0.4995	0.0083	0.0032	0.3552
Ca Dn	0.5845	0.0359	0.7026	0.0661	0.0638	0.0877

***Supplemental Table 7*: 2-way ANOVA comparing individual and combined effects of ablated TGFβ signaling, HCD, and HFD diet on trabecular and cortical bone parameters of control and TβRII$^{ocy-/-}$ mouse bones.**

p values						
	**TGFβ factor**	**HCD factor**	**TGFβ * HCD Interaction**	**TGFβ factor**	**HFD factor**	**TGFβ * HFD Interaction**
**Distal femur**						
Tb.BV/TV (%)	0.0172	0.2534	0.0172	<0.0001	0.3752	0.0261
Tb. N (1/mm)	<0.0001	0.3106	0.0259	<0.0001	0.0578	0.0026
Tb. Th (mm)	0.0002	0.6229	0.0028	0.0361	0.6015	0.0361
Tb. Sp (mm)	<0.0001	0.2540	0.0135	<0.0001	0.3334	0.0029
SMI	0.5792	<0.0001	0.0099	0.6863	<0.0001	0.2101
Tb. tBMD (mg HA/cm^3)	<0.0001	0.1165	0.0002	0.0011	0.1874	0.0042
Conn-Den	0.1888	<0.0001	0.0460	0.0146	<0.0001	0.0010
**Femoral midshaft**						
Ct. BA/TA (%)	0.0495	0.0007	0.0088	<0.0001	<0.0001	0.0002
Ct. Th (mm)	0.0206	<0.0001	0.0924	0.1483	<0.0001	0.0018
Ct. tBMD (mg HA/cm^3)	<0.0001	0.2196	0.7577	0.0025	0.0511	0.0405

***Supplemental Table 8*: 2-way ANOVA comparing individual and combined effects of ablated TGFβ signaling, HCD, and HFD diet on flexural strength parameters of control and TβRII$^{ocy-/-}$ mouse bones.**

p values						
**Flexural strength parameters**	**TGFβ factor**	**HCD factor**	**TGFβ * HCD Interaction**	**TGFβ factor**	**HFD factor**	**TGFβ * HFD Interaction**
Stiffness (N-mm^2)	<0.0001	0.5548	0.2926	0.0006	0.2128	0.1746
Yield load (N)	0.0005	<0.0001	0.4990	0.0014	<0.0001	0.3045
Post-yield displacement (mm)	0.7577	0.0003	0.7209	0.1351	0.0529	0.1237
WorkFx (N-mm)	0.2182	0.3947	0.8935	<0.0001	0.2723	0.0936
Ultimate force (N)	0.0003	0.2655	0.3635	<0.0001	0.0013	0.0948
FxForce (N)	0.0038	0.0182	0.1410	0.0007	0.0328	0.0241
Yield Stiffness (N/mm)	0.0006	0.3871	0.2432	0.0015	0.2607	0.1636
Elastic Modulus (GPa)	0.1583	<0.0001	0.0019	0.7832	0.0049	<0.0001
Yield Stress (Mpa)	0.0693	0.1270	0.0240	0.0916	0.0007	0.0314
Ultimate stress (Mpa)	0.2528	<0.0001	0.0045	0.0024	<0.0001	<0.0001

Supplemental Figures.

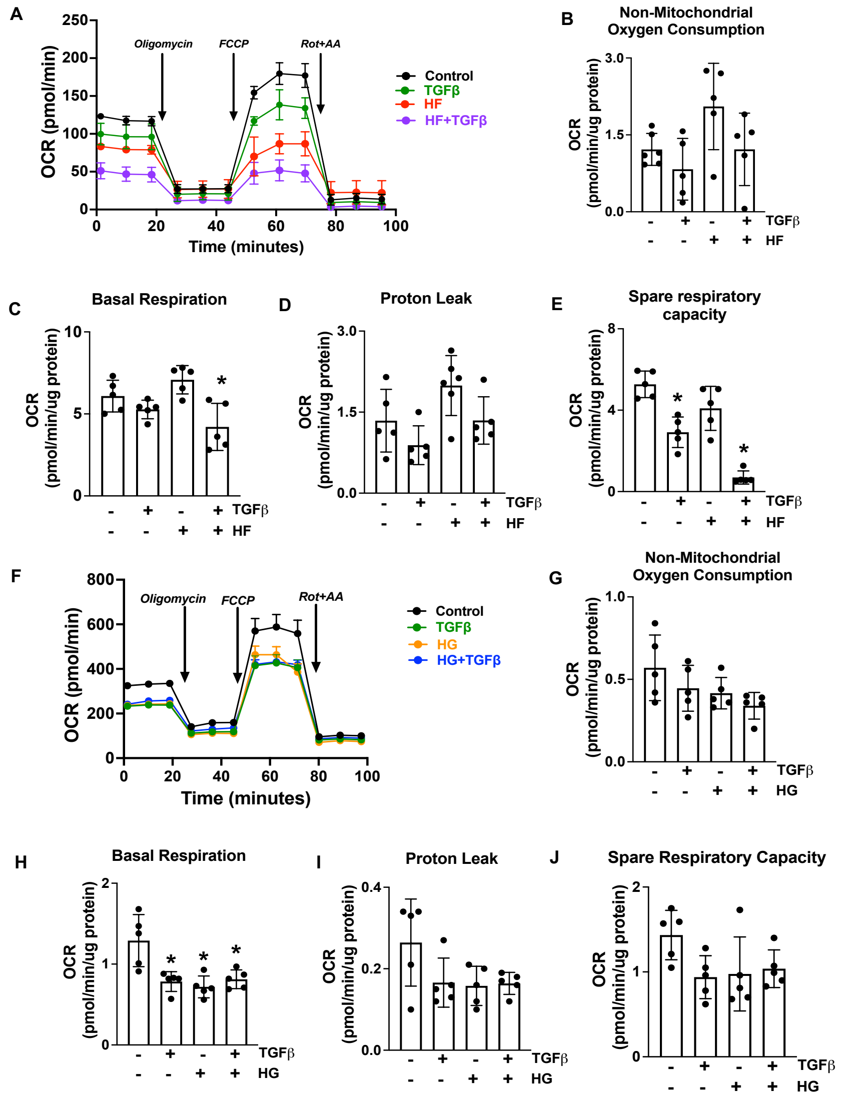

Figure S1. Hyperlipidemia, hyperglycemia, and TGFβ signaling alter osteocyte intrinsic cellular metabolism. Undifferentiated OCY454 cells were exposed to hyperlipidemia (HF, palmitate-100 μm, oleate-200 μm, linoleate-200 μm) or hyperglycemia (HG, 25 mM) in the presence or absence of TGFβ (5 ng/ml) for 24 hr. Bar graphs showing quantified cell-normalized mitochondrial OCR from stress tests in HF (A-F) and HG (G-K) treated OCY454 cells. Data are representative of 3 independent experiments performed with N=5 technical replicates has been expressed as mean ± SD, $^{*}p < 0.05$ different from untreated group, $^{\#}p < 0.05$ different from TGFβ treated group, $^{\$}p < 0.05$ different from HF or HG treated groups in the respective experiments.

Statistical significance was calculated using two-way ANOVA with Newman-Kuels multiple posthoc correction.

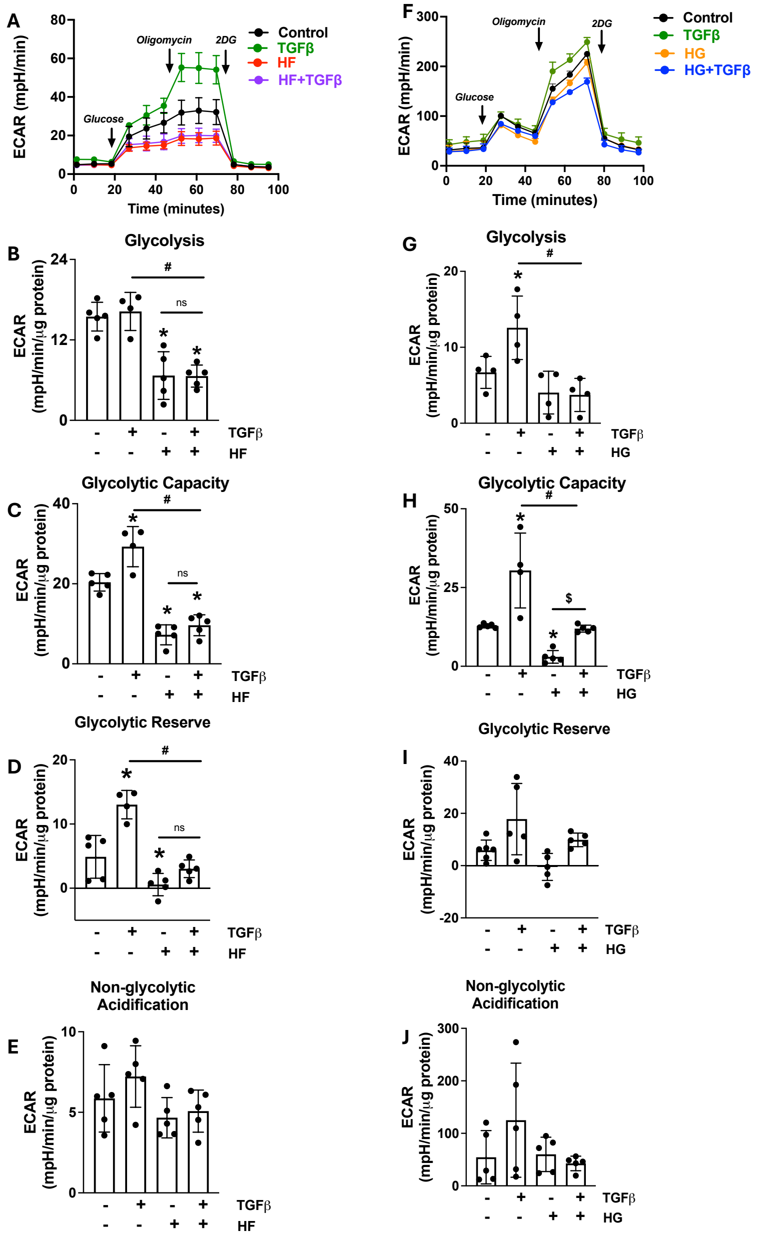

Figure S2. Hyperglycemia, hyperlipidemia, and TGFβ signaling impact osteocytic glycolysis. Undifferentiated OCY454 cells were exposed to hyperlipidemia (HF, palmitate-100 µm, oleate-200 µm, linoleate-200 µm) or hyperglycemia (HG, 25 mM) in the presence or absence of TGFβ (5 ng/ml) for 24 hr. Bar graphs showing quantified cell-normalized glycolytic ECAR in HF

(A-C) and HG (D-F) with and without TGFβ supplementation. Data are representative of 3 independent experiments performed with N=5 technical replicates has been expressed as mean ± SD, *p < 0.05 different from untreated group, #p < 0.05 different from TGFβ treated group, $p < 0.05 different from HF or HG treated groups in the respective experiments. Statistical significance was calculated using two-way ANOVA with Newman-Kuels multiple posthoc correction.

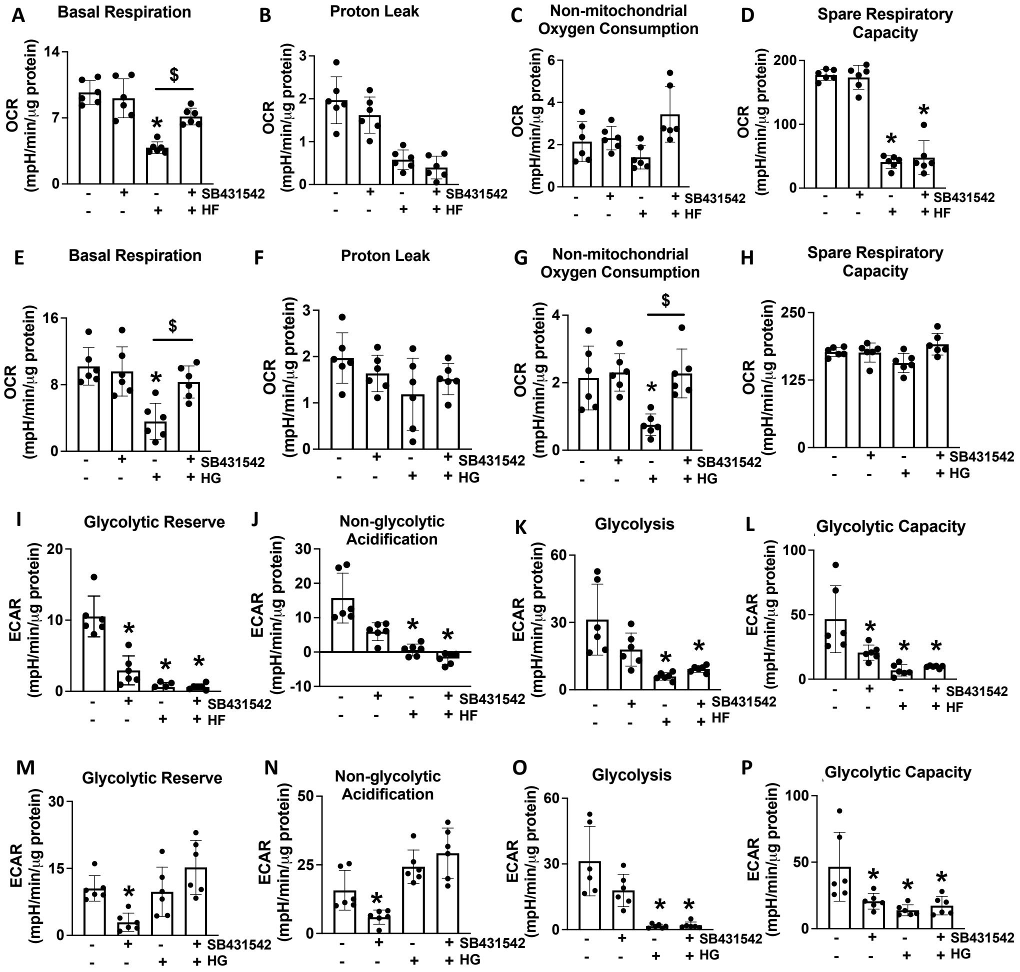

Figure S3. Blockade of TGFβ signaling to some extent mitigates effects of hyperlipidemia and hyperglycemia on osteocyte intrinsic cellular metabolism. Undifferentiated OCY454 cells were exposed to hyperlipidemia (HF, palmitate-100 μm, oleate-200 μm, linoleate-200 μm)

or hyperglycemia (HG, 25 mM) in the presence or absence of TGFβ receptor I-kinase inhibitor, SB431542 (10 μM) for 24 hr. Bar graphs showing quantified cell-normalized mitochondrial OCR from stress tests in HF (A-D) and HG (E-H) treated OCY454 cells. Effects of HF (I-L) and HG (M-P) on the glycolytic pathway are independent of TGFβ blockade by SB431542. Data are representative of 3 independent experiments performed with N=5 technical replicates has been expressed as mean ± SD, *p < 0.05 different from untreated group, #p < 0.05 different from TGFβ treated group, $p < 0.05 different from HF or HG treated groups in the respective experiments. Statistical significance was calculated using two-way ANOVA with Newman-Kuels multiple posthoc correction.

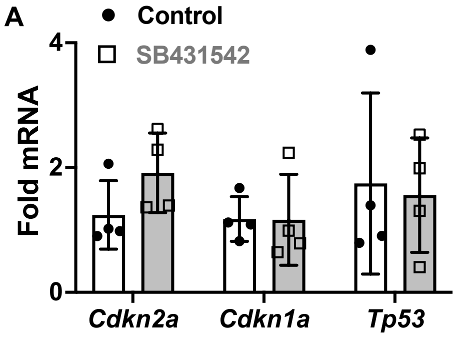

Figure S4. Inhibition of TGFβ signaling in vitro in osteocytes does not impact the transcript levels of senescent markers p16^{ink4a}, p21^{cip}, and p53. Differentiated OCY454 cells were treated with TGFβ receptor I-kinase inhibitor, SB431542 (10 μM) for 72 hr. qPCR analysis shows similar induction in the mRNA levels of senescence markers, Cdkn2a (p16^{ink4a}), Cdkn1a (p21^{cip}), and p53 (Tp53) in differentiated OCY454 cells treated with or without SB431542. 18S RNA was used as a housekeeping gene. N=4 replicates/group across 3 independent experiments and statistics were conducted using Student's t-test.

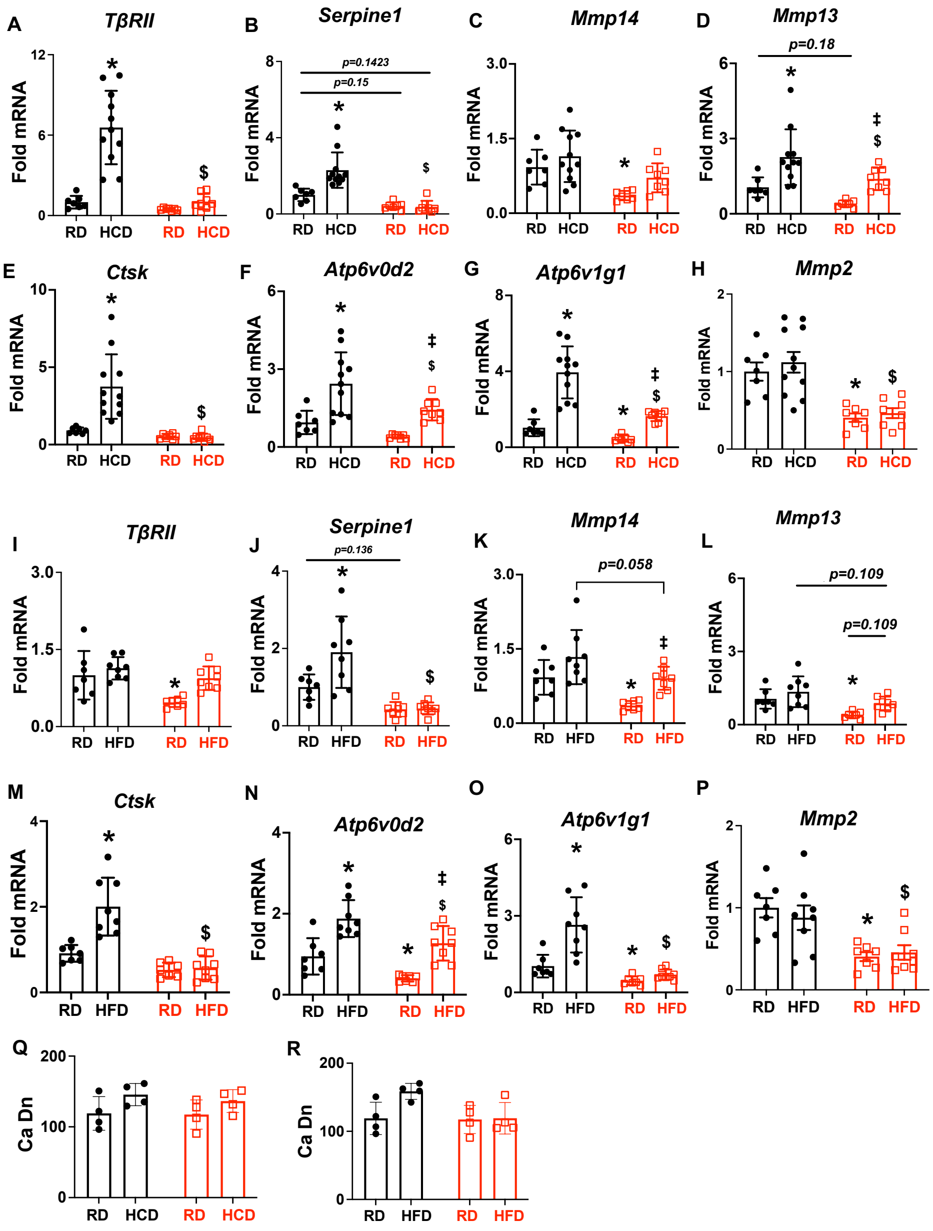

Figure S5. HCD and HFD diets impact PLR gene expression or vascular canal density in cortical bone. (A-P) qRT-PCR of cortical bone-derived RNA from RD, HCD, or HFD fed 30-week-old control and TβRII$^{ocy-/-}$ mice were used for in vivo assessment of PLR genes. mRNA levels of PLR genes were normalized to that of 18sRNA, and fold change mRNA levels has been shown as mean ± SD. N=7-11 mice/ groups were used. These figures are made from data shown in Fig. 5A-B and 6K-O and compare the effects of diet RD vs. HCD or RD vs. HFD with TβRII disruption and the interaction between diet and TβRII disruption. **(Q, R)** SRμCT detected no

differences in vascular canal density in tibial cortical bones of RD, HCD, or HFD-fed 30-week-old control and TβRII$^{ocy-/-}$ mice. A sample size of N=4 mice/group was used for SRμCT assessment. *$p<0.05$ different from RD-fed control mice, $^{\#}p<0.05$ different from HCD-fed control mice. All statistical differences were calculated with two-way ANOVA, with Newman-Kuels multiple post-hoc correction. RD, standard chow; HCD, low-fat diet, high carbohydrate diet; HFD, high-fat diet.

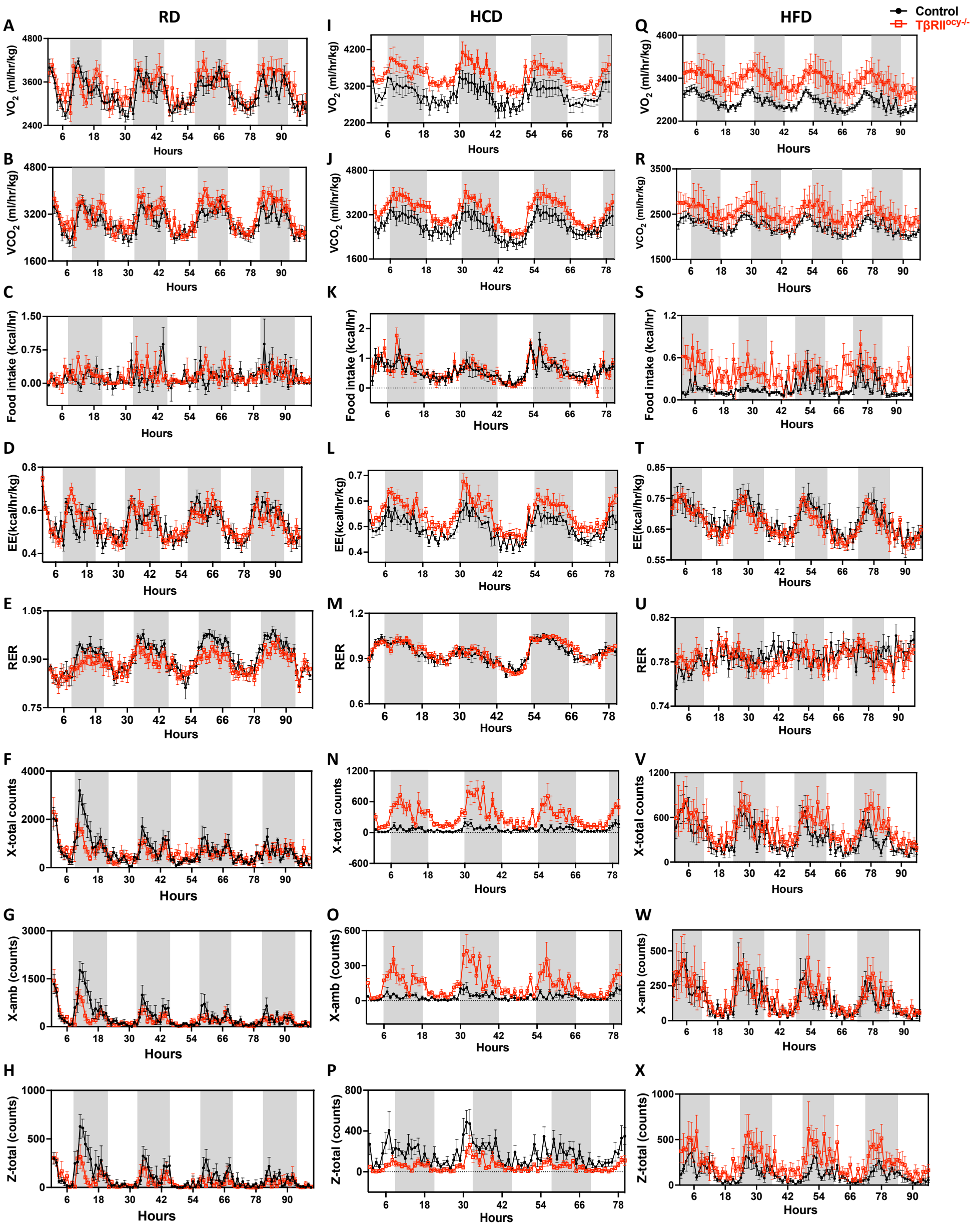

Figure S6. TGFβ signaling disruption in osteocytes protects against HCD and HFD induced changes in energy expenditure, activity, and food intake in mice. Using Oxymax CLAMS, Oxygen consumption (VO2) and Carbon Dioxide production (VCO2), energy expenditure (EE), respiratory exchange ratio (RER), food intake, and activity (X-amb and Z-count) were analyzed by indirect calorimetry in male control and TβRII$^{ocy-/-}$ mice fed RD **(A-H)**, HCD **(I-P)**, or HFD **(Q-X)** diet for 18-weeks. Results are mean ± SEM of 6-8 animals/group. Statistical significance was assessed by two-tailed Student's t-test, * p<0.05.

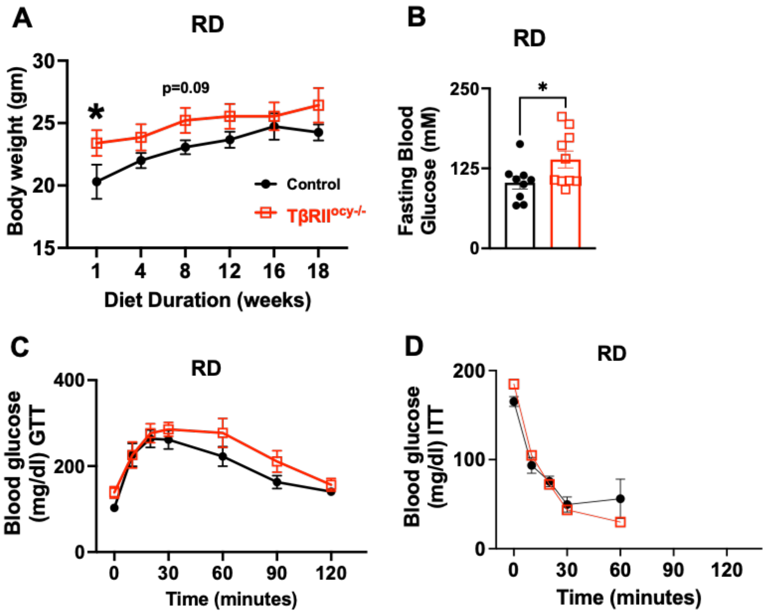

Figure S7. TβRII$^{ocy-/-}$ female mice show higher body weight and fasting blood glucose levels. Body weight **(A)** and blood glucose levels **(B)** following an overnight fast in female control and TβRII$^{ocy-/-}$ mice fed regular chow diet (RD) is shown. Intraperitoneal glucose tolerance test (GTT) **(C)** and insulin tolerance test (ITT) **(D)** were performed at the end of diet in male control and TβRII$^{ocy-/-}$ mice fed regular chow diet (RD). Results are mean ± SEM of 10 animals/group. Statistical significance assessed by two-tailed Student's t-test, * p<0.05 denotes a significant difference from the control group on the same diet.
