# Supplementary figures and images for: High-fat and high-carbohydrate diets increase bone fragility through TGF-β–dependent control of osteocyte function
Source: JCI Insight. 2024 Jul 9;9(16):e175103. doi: 10.1172/jci.insight.175103 (PMC11343608; doi:10.1172/jci.insight.175103)

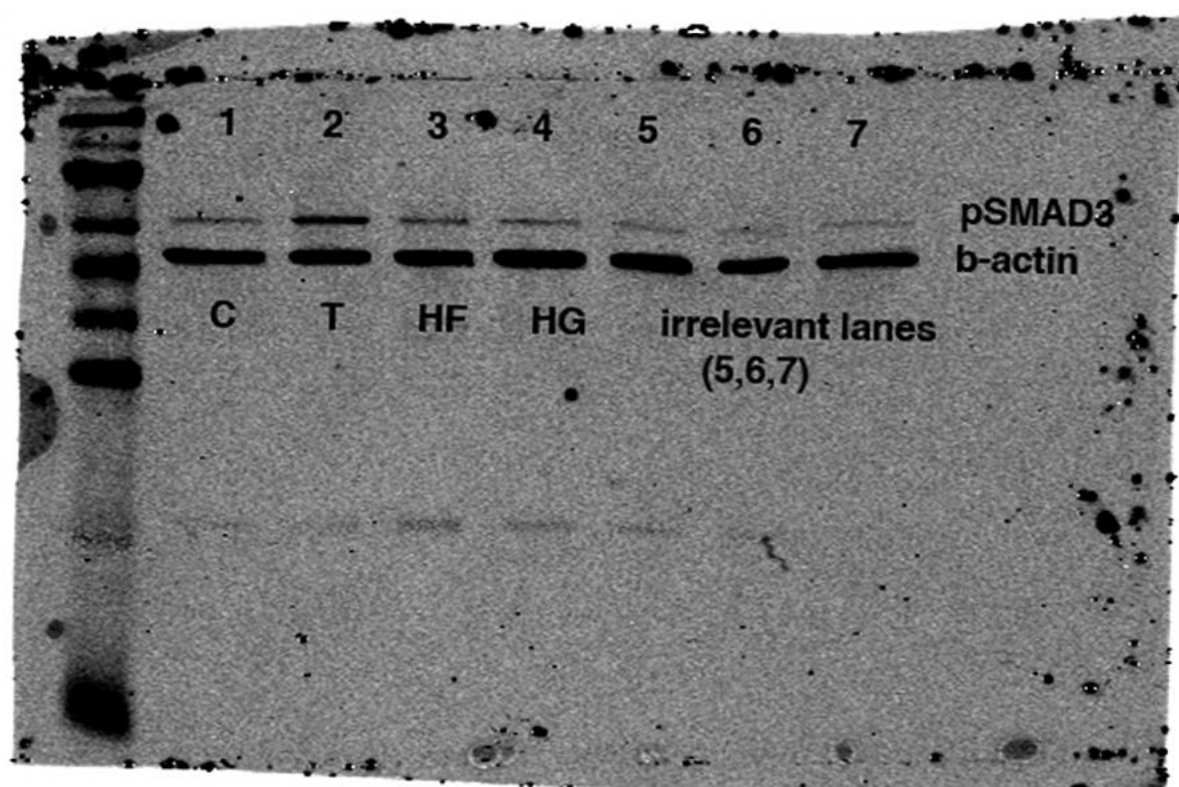

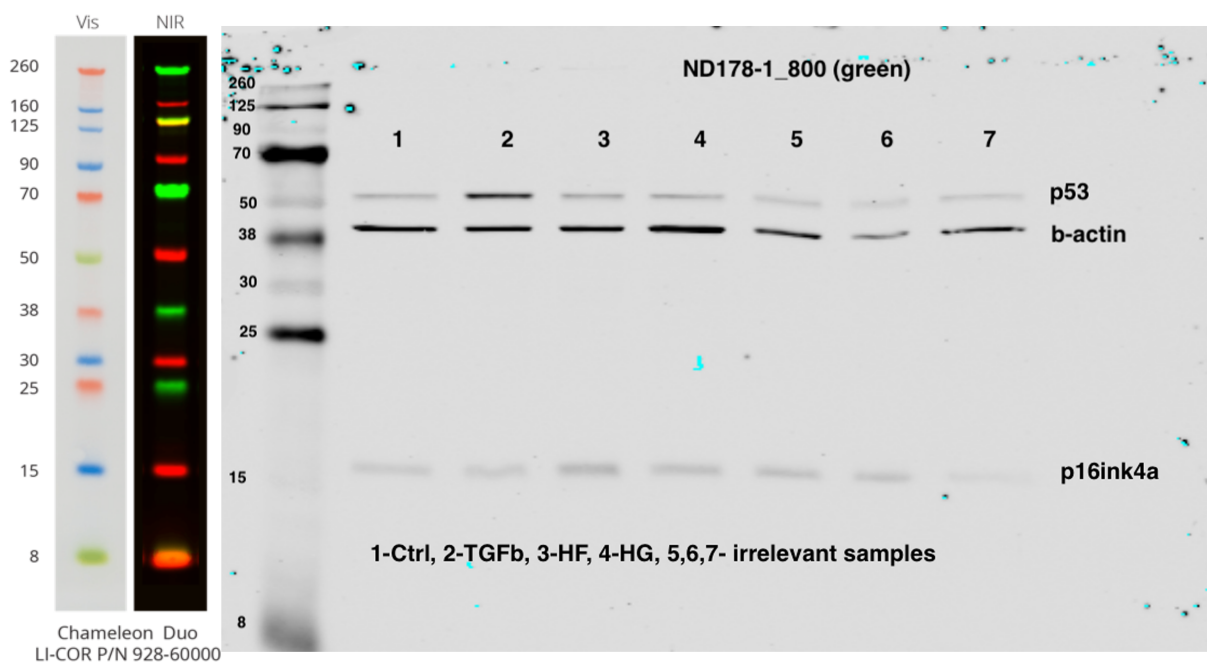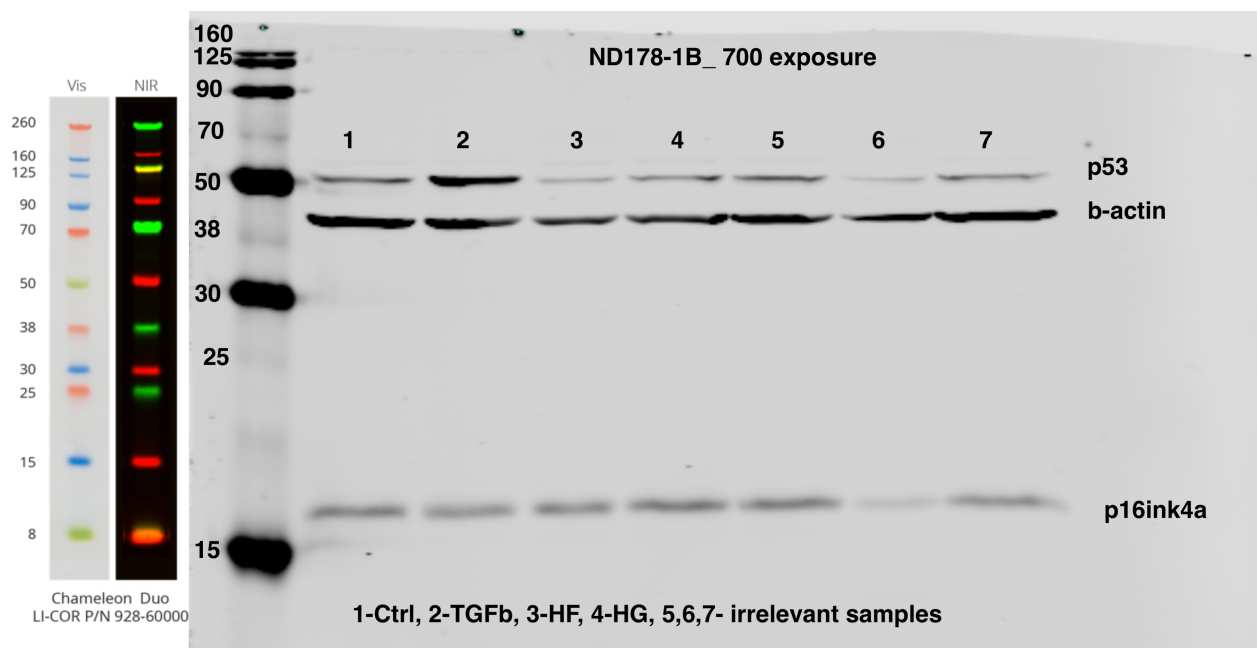

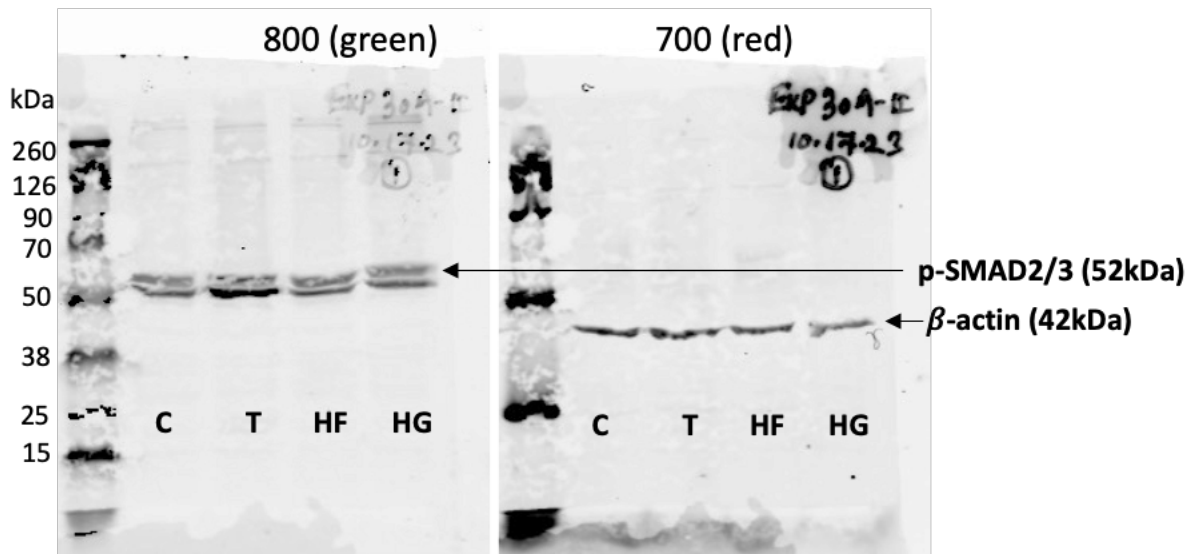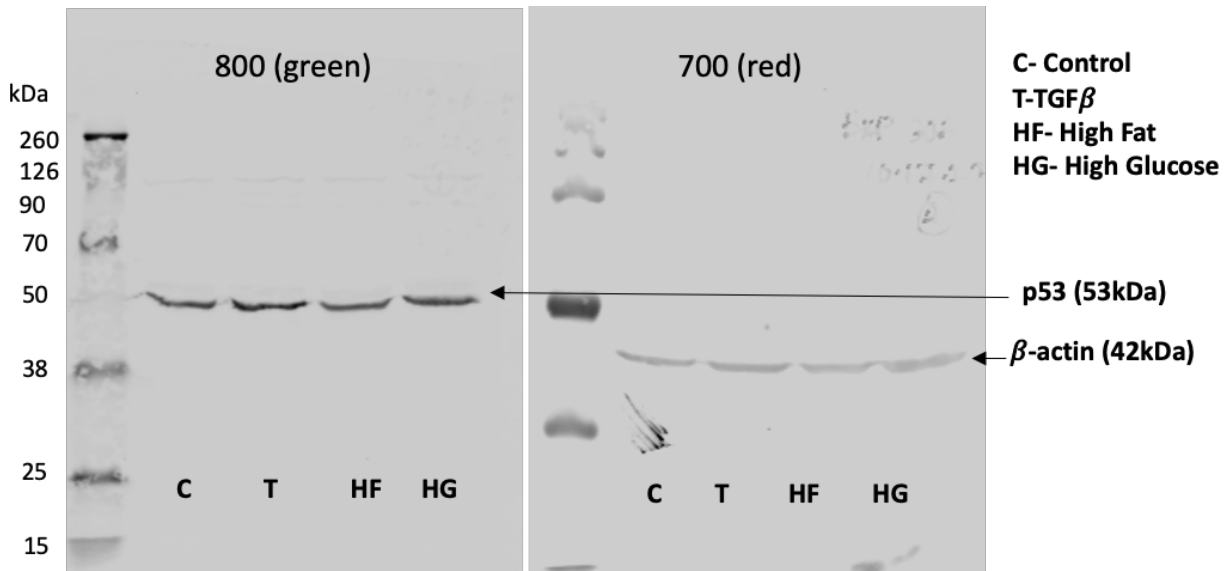

Supplement: Unedited blot and gel images [file jciinsight-9-175103-s204.pdf]
